# Revealing brain connectivity: graph embeddings for EEG representation learning and comparative analysis of structural and functional connectivity

**DOI:** 10.3389/fnins.2023.1288433

**Published:** 2024-01-08

**Authors:** Abdullah Almohammadi, Yu-Kai Wang

**Affiliations:** ^1^School of Computer Science, Faculty of Engineering and Information Technology, University of Technology Sydney, Sydney, NSW, Australia; ^2^College of Computer Science and Engineering, Taibah University, Madinah, Saudia Arabia

**Keywords:** brain connectivity, Graph Convolutional Neural Network, structural connectivity, adjacency matrix, functional connectivity, PLV, motor imagery, MI-EEG

## Abstract

This study employs deep learning techniques to present a compelling approach for modeling brain connectivity in EEG motor imagery classification through graph embedding. The compelling aspect of this study lies in its combination of graph embedding, deep learning, and different brain connectivity types, which not only enhances classification accuracy but also enriches the understanding of brain function. The approach yields high accuracy, providing valuable insights into brain connections and has potential applications in understanding neurological conditions. The proposed models consist of two distinct graph-based convolutional neural networks, each leveraging different types of brain connectivities to enhance classification performance and gain a deeper understanding of brain connections. The first model, Adjacency-based Convolutional Neural Network Model (Adj-CNNM), utilizes a graph representation based on structural brain connectivity to embed spatial information, distinguishing it from prior spatial filtering approaches dependent on subjects and tasks. Extensive tests on a benchmark dataset-IV-2a demonstrate that an accuracy of 72.77% is achieved by the Adj-CNNM, surpassing baseline and state-of-the-art methods. The second model, Phase Locking Value Convolutional Neural Network Model (PLV-CNNM), incorporates functional connectivity to overcome structural connectivity limitations and identifies connections between distinct brain regions. The PLV-CNNM achieves an overall accuracy of 75.10% across the 1–51 Hz frequency range. In the preferred 8–30 Hz frequency band, known for motor imagery data classification (including α, μ, and β waves), individual accuracies of 91.9%, 90.2%, and 85.8% are attained for α, μ, and β, respectively. Moreover, the model performs admirably with 84.3% accuracy when considering the entire 8–30 Hz band. Notably, the PLV-CNNM reveals robust connections between different brain regions during motor imagery tasks, including the frontal and central cortex and the central and parietal cortex. These findings provide valuable insights into brain connectivity patterns, enriching the comprehension of brain function. Additionally, the study offers a comprehensive comparative analysis of diverse brain connectivity modeling methods.

## 1 Introduction

In the realm of establishing a brain-computer interface (BCI) with practical applications in rehabilitating stroke patients (López et al., 2019), an essential focus lies in the precise classification of motor imagery. Electroencephalographic (EEG) signals have emerged as a prominent physiological cue for constructing a BCI system, offering the advantage of non-invasively capturing the electrical activity within the cortex through easily recorded scalp measurements. Consequently, researchers have extensively explored EEG-based BCIs, drawn by their low clinical implications, portability, and cost-effectiveness.

Although significant progress has been made in EEG-based motor imagery (MI) classification in recent years, most of the publications focus on the subject-dependent scenario. In this scenario, training and testing data come from the same group of individuals, and a calibration session is required for each new user. This limits the scalability and applicability of BCI devices. Overcoming the subject-independent challenge is crucial, but it is difficult due to the differences in EEG signals between individuals (Suk and Lee, 2012). Conventional approaches to EEG analysis use handcrafted features and machine learning algorithms, with power spectral density (PSD) being one of the most popular features. When examining the PSD patterns of motor imagery EEG data, researchers often observe event-related synchronization/desynchronization (ERS/ERD), indicating an increase or decrease in EEG power in certain frequency bands (Hamedi et al., 2016). Nevertheless, not all EEG electrodes provide unique data regarding PSD properties.

The most effective EEG nodes are typically selected through an EEG channel selection method (Yang et al., 2017). The primary motor cortex is a crucial brain region for classifying motor imagery tasks, and under the widely used 10–20 EEG system placement, the three channels C3, C4, and Cz have been identified as the most informative channels for capturing and classifying motor-related brain activity. Nevertheless, limitations arise when employing handcrafted features. First of all, earlier research has conflicting information on the range of different frequency bands (Reilly, 2013; Nayak and Anilkumar, 2020). Secondly, the number of useful EEG nodes chosen by a channel selection algorithm is often determined by the expertise and experience of an expert. Thirdly, isolating all phases in conventional works is inefficient and may waste time. Furthermore, it removes the possibility that distinct steps could encourage each other in the feature learning process. While robust predictive models have been applied to handcrafted features to improve performance (Ieracitano et al., 2019), human-designed features may overlook crucial information within the raw EEG data (Jiao et al., 2018). In contrast, deep learning algorithms can effectively learn underlying information across different subjects. Substantial efforts have been dedicated to developing EEG analysis algorithms that incorporate deep learning techniques, with promising outcomes (Bashivan et al., 2015; Lawhern et al., 2018; Zhang et al., 2018b; Zhang P. et al., 2018; Essa and Kotte, 2021). A compact Convolutional Neural Network (CNN) is introduced in Lawhern et al. (2018), which shows success on many different types of EEG paradigms. Additionally, Recurrent Neural Networks (RNNs) with Long Short-Term Memory (LSTM) cells have been suggested in Zhang et al. (2018b) to effectively exploit temporal dynamics. Moreover, several publications (Bashivan et al., 2015; Lawhern et al., 2018; Zhang et al., 2018b; Zhang P. et al., 2018; Essa and Kotte, 2021) have integrated deep learning techniques with traditional spectral features. Despite deep learning's success in EEG analysis, only a limited number of studies have constructed a motor imagery classification model that can generalize to new subjects (Riyad et al., 2019; Zhu et al., 2019).

In the field of EEG-based MI classification, a variety of studies have emerged, each offering unique insights and techniques to enhance the field. While earlier approaches have relied on handcrafted features to enhance performance, it has become evident these methods may inadvertently overlook vital information embedded in raw EEG data. Consequently, deep learning algorithms have gained traction for their ability to effectively uncover latent patterns across diverse subjects. A notable study by Zhao et al. (2019) introduces a groundbreaking 3D representation of EEG data for MI classification. This innovative approach preserves both spatial and temporal information, harnessing the power of a multi-branch 3D CNN to extract MI-related features. The fusion of this 3D EEG representation with the multi-branch 3D CNN yields remarkable results, including a substantial 50% reduction in subject-based variability, all while utilizing only nine electrodes. In a related vein, Liao et al. (2020) explore the “EEG-as-image” paradigm for MI classification. Their study introduces three distinct deep learning models, each employing different spatial convolution strategies. Impressively, their global model achieves a classification accuracy of 74.6%, surpassing other models by 2.8% and 1.4%, highlighting the significant impact of spatial filters on classification accuracy. Alwasiti et al. (2020) take a distinct approach by leveraging deep metric learning to classify MI EEG signals for BCI applications. They employ a triplet network and Stockwell Transform to navigate the challenges posed by inter-individual variability in MI-BCI EEG signals. Their approach shows promise, achieving convergence with a minimal number of training samples. Collazos-Huertas et al. (2020) aim to enhance the interpretability of MI classification using CNNs applied to EEG data. They explore two 2D feature extraction methods and incorporate spatial dropping to remove irrelevant brain regions. Their framework improves classification accuracy and uncovers spatially relevant electrodes for MI tasks, albeit with some challenges related to dataset size and potential overfitting. Fan et al. (2021) introduce QNet, a novel deep learning network that incorporates a specially designed attention module known as 3D-AM. This module enables automatic learning of electrode and time selection, leading to QNet outperforming existing methods in EEG MI classification tasks. QNet achieves results on the Physionet dataset, with an average cross-validation accuracy of 65.82% for four classes, 74.75% for three classes, and 82.88% for two classes.

Amin et al. (2021) harness the power of deep learning with an attention-based CNN model. Their Multi-CNN Feature Fusion approach addresses inter-subject and inter-session variability, achieving superior performance compared to existing methods. Furthermore, on the BCI-IV 2a dataset, the proposed model achieved an accuracy of 74.70%. These studies collectively highlight the increasing impact of deep learning techniques in EEG-based MI classification. They showcase innovative approaches that offer promise for improved accuracy and broader applicability across subjects and contexts.

This paper presents graph-based convolutional neural network models that incorporate both structural and functional connectivity. These models have been rigorously validated using a benchmark EEG motor imagery dataset, demonstrating their proficiency. They outperform various comparative methods, emphasizing the importance of understanding the intricate relationship between neural connections.

The primary contributions of this work can be summarized as follows:

- Novel graph embedding techniques for EEG signals: This section introduces innovative graph embedding techniques to represent EEG signals, encompassing both structural and functional brain connectivity aspects. It effectively reduces complexity while preserving critical brain connectivity information and revealing hidden relationships between brain regions. Additionally, it captures intricate brain interactions during cognitive tasks, providing a comprehensive exploration of brain networks. The graph representation extracts meaningful features that enhance the accuracy of cognitive state and brain activity classification.
- Two deep learning methodologies for EEG motor imagery classification: This section presents two distinct deep learning approaches for EEG motor imagery classification. In the first approach, Adj-CNNM, EEG spatial information is represented using graph embedding with an Adjacency Matrix, maintaining independence across individuals and tasks to comprehensively understand brain network organization. The second approach, PLV-CNNM, utilizes Phase Locking Value (PLV) within a graph embedding representation, enabling the analysis of functional connectivity patterns. These approaches offer unique perspectives on leveraging deep learning for EEG motor imagery classification, considering both structural and functional connectivity separately for more informed and accurate classification.
- Comprehensive benchmark testing: Rigorous testing was conducted on the widely recognized benchmark dataset BCI-IV2a, providing a robust evaluation of the proposed methodologies.
- Comparative analysis of brain connectivity methods: This section includes a comparative analysis of various brain connectivity methods on motor imagery tasks, covering both structural and functional connectivity. It reveals distinct advantages and limitations of different approaches.
- In-depth model interpretation: This section offers in-depth insights and discussions on model interpretation for both structural and functional brain connectivity, enhancing the understanding of the underlying cognitive processes.

## 2 Related work

### 2.1 EEG motor imagery classification

The classification of motor imagery using EEG is a fundamental aspect of various BCIs, and several techniques have been proposed to address this challenge. One of the most well-known and effective feature extraction algorithms used in motor imagery EEG classification is the Common Spatial Pattern (CSP) (Xygonakis et al., 2018). CSP is a feature extraction technique that utilizes spatial filters to enhance the discriminative power between two distinct MI classes by identifying a linear combination of EEG channels (Ramoser et al., 2000). There have been many reports of efforts to improve CSP and significantly enhance its performance (Ang et al., 2008; Lotte and Guan, 2010). Filter Bank CSP (FBCSP) is a popular variant of CSP that overcomes the original method's limitation, which depends on a single frequency band, by adapting it to work across multiple bands and selecting subject-specific features using a feature selection technique (Ang et al., 2008). FBCSP was the most advanced technique in motor imagery EEG classification and achieved remarkable success (Ang et al., 2012). Many studies that use EEG motor imagery employ traditional classifiers such as Support Vector Machines (SVM) and linear discriminant analysis (LDA) (Schlögl et al., 2005; Ang et al., 2008). Researchers have utilized deep learning techniques to improve motor imagery classification due to its higher performance and end-to-end structure. In Schlögl et al. (2005), an improved performance over FBCSP is proposed, which involves a crop training technique and a well-designed CNN. A lightweight CNN is developed in Lawhern et al. (2018), demonstrating performance comparable to state-of-the-art approaches in various BCI paradigms, including motor imagery and P300. To extract temporal information, RNN is widely used in motor imagery classification. According to Zhang et al. (2018a), fuzzy measures can be optimized by fusing CNN and RNN with a fuzzy integral and using a reinforcement learning approach. Deep learning models' effectiveness can also be enhanced by using feature engineering. Power spectral features are commonly used to classify motor imagery due to their ability to discriminate, as shown in prior research (Herman et al., 2008). In Pérez-Zapata et al. (2018), a combination of CNNs and PSD features is proposed for motor imagery classification, achieving promising results.

### 2.2 Graph Neural Network and brain connectivity

Graph Neural Networks (GNNs) have revolutionized the study of brain connectivity, providing a powerful approach to understanding both structural and functional aspects of brain networks. Unlike traditional methods that rely on feature engineering, GNNs can directly learn and represent reasoning graphs from massive graph datasets, making them ideal for brain connectivity analysis (Zhou et al., 2020).

Structural connectomes, representing the neural connections among different brain regions, have been a focus of GNN applications in brain connectivity analysis. To effectively analyze structural connectomes, researchers have developed the Relational Graph Neural Network (RGNN). RGNN is specifically designed to work with structured brain data, restricting message passing through node and edge layers to model information flow effectively (Shanthamallu et al., 2019). Utilizing the Human Connectome Project (HCP) data, RGNN accurately predicts brain region volumes and meta-information, outperforming traditional methods (Shanthamallu et al., 2019).

Functional connectivity, which measures the temporal correlations between brain regions, is crucial in understanding brain dynamics. GNNs have also made significant contributions to functional connectivity analysis. The Multi-resolution Graph Neural Network (MGNN) offers a novel approach to study brain diseases related to functional connectomes (Ma et al., 2019). By utilizing adaptive graph transformation and convolutional neural networks, MGNN achieves impressive accuracy in classifying graphs from structural brain connectivity datasets (Ma et al., 2019).

In addition to structural and functional connectivity, GNNs have been instrumental in advancing BCI research. Studies have utilized graph metrics to classify MI-based BCIs. One such study proposed a novel approach to classify MI-based BCI by utilizing individual features acquired from graph theory applied to functional connectivity measures (Santamaria and James, 2016). By translating connectivity measures into complex networks, the study achieved promising results with over 80% overall performance and up to 91.1% accuracy in some MI tasks.

This work proposes applying the graph theory to characterize the spatial and functional interconnections among EEG nodes by leveraging both structural and functional brain connectivity. With the integration of GNNs in brain connectivity analysis, researchers are better equipped to comprehend the intricate interplay of neural interactions, paving the way for new discoveries in neuroscience. From predicting brain region volumes to classifying brain diseases and exploring Motor Imagery-based BCIs, the applications of GNNs in brain connectivity analysis hold great promise for advancing the understanding of the brain's complexities. As research in this field continues, GNNs are set to shape the future of neuroscience, driving breakthroughs in brain-related fields and fostering new avenues for innovative brain-computer interfaces and neurological disorder detection.

## 3 Methodology

### 3.1 Dataset and pre-processing

**Figure 1:**
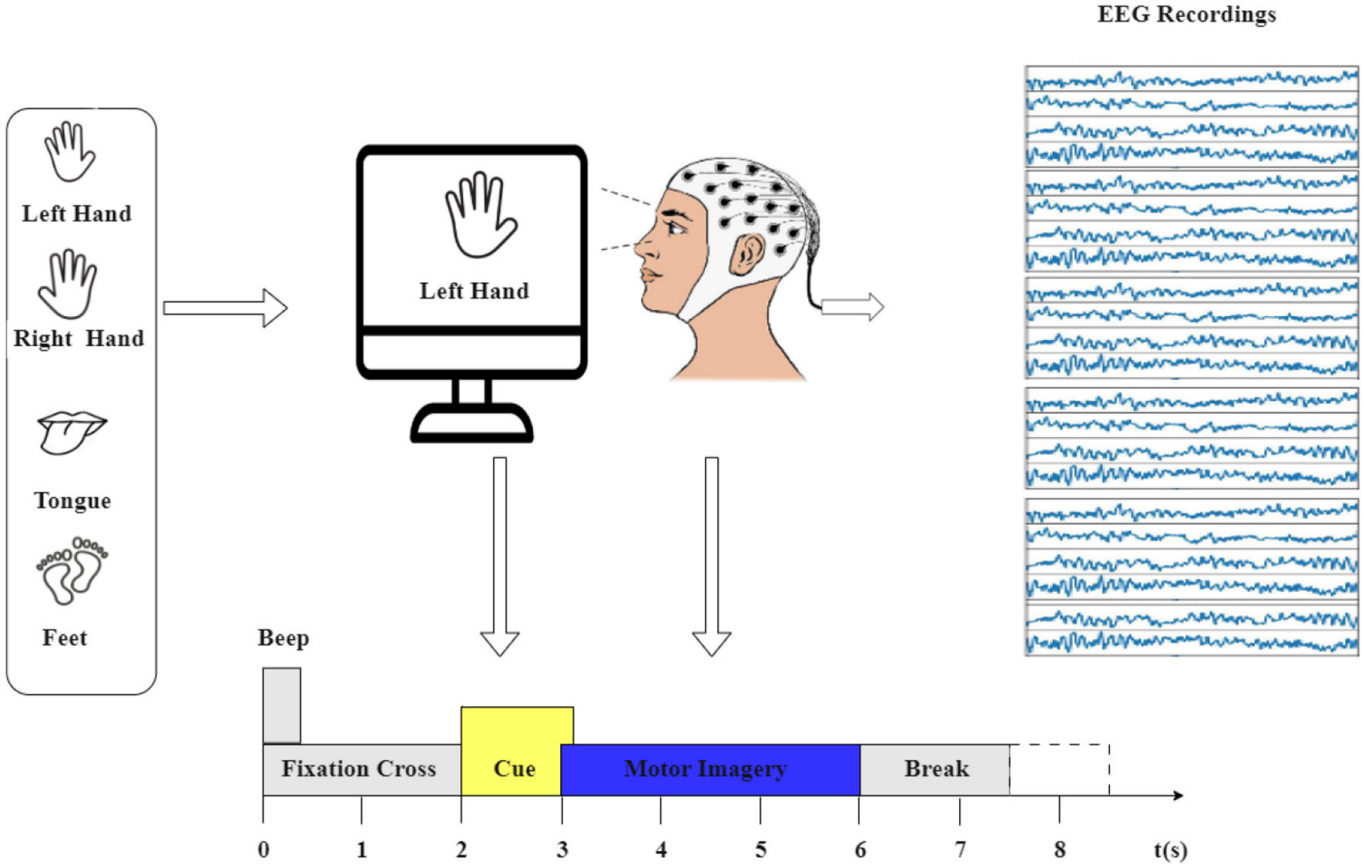
The experimental paradigm was divided into four sections: (1) A 2-second period with eyes open, where participants focused on a fixation cross displayed on the screen after a warning tone. (2) A visual cue depicting a different motor imagery (MI) task appeared on the screen for 1.25 s. (3) Participants were instructed to perform the motor imagery task until the conclusion of the MI cue at *t* = 6 s. (4) Following this, a brief intermission followed.

The motor imagery experiment process is depicted in Figure 1. The dataset used in this study is the BCI Competition IV-2a (Brunner et al., 2008), a widely recognized EEG benchmark dataset. It captures 22 EEG signals sourced from nine healthy participants engaged in two distinct sessions over separate days. Each session consists of 288 trials involving four different motor imagery tasks: imagining the movement of the left hand (class 1), right hand (class 2), both feet (class 3), and tongue (class 4). The EEG signals were recorded at a sampling rate of 250 Hz and were bandpass-filtered between 0.5 and 51 Hz.

The first session's 288 trials were used for training, and the second session's 288 trials were used for testing in the original dataset. However, the dataset underwent pre-processing using EEG-lab, with the extracted epochs lasting 4 s, aimed at capturing the duration of motor imagery tasks. This focus is guided by the cue signaling the start of a task, which appears at *t* = 2 s, prompting subjects to engage in the motor imagery task until the fixation cross disappears at *t* = 6 s. Importantly, this time span constitutes 4 s.

This study used data from the first session, resulting in a total of 2,592 trials for the nine participants (9*288). Consequently, the culmination of EEG data across all trials yields the final shape of (22, 1,000, 2,592), signifying the channels, time points, and trials, respectively.

### 3.2 Graph representation based on structural connectivity

**Figure 2:**
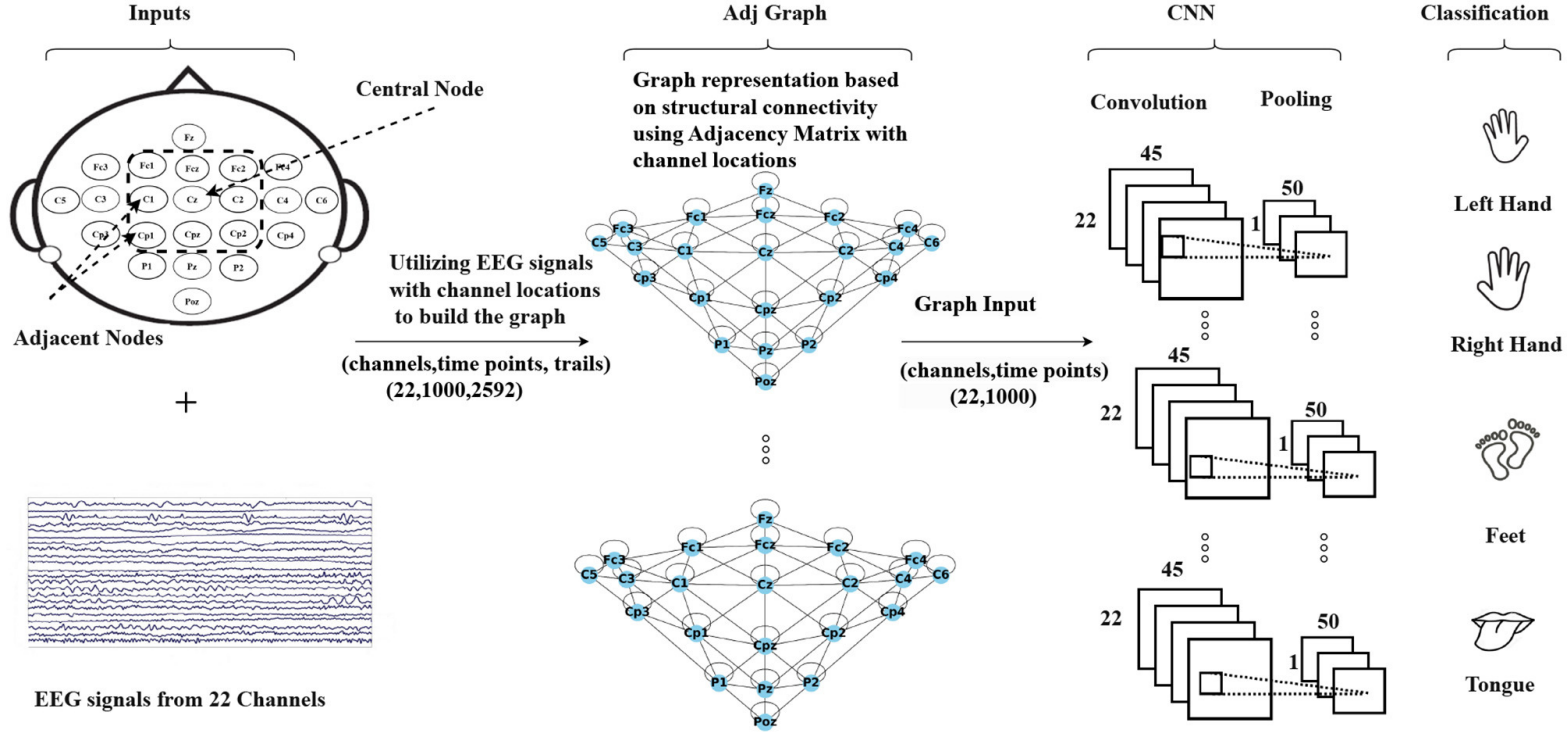
An illustration of the Graph Convolutional Neural Network approach based on structural connectivity (adjacency matrix) to classify MI-EEG data, where structural connectivity represents the anatomical pathways that connect different brain regions, forming a network for information exchange.

The methodology is summarized in Figure 2, which illustrates the process. The first step involves creating a graph representation to embed the EEG signals. These graph-encoded EEG signals are then fed into the neural network. The entire framework represents an end-to-end model that can undergo training utilizing standard back-propagation techniques.

The Adj-CNNM is a convolutional neural network that uses graph representations of EEG nodes to effectively learn spatial information. This approach can help address the issue of subject-independence in EEG-based motor imagery classification. The first step is to create an EEG graph based on the locations of EEG electrodes, which represents the connections between electrodes with accurate spatial information. The suggested graph embedding technique is more flexible and reliable for new subjects as it does not rely on specific subjects, unlike previous spatial filtering methods.

#### 3.2.1 EEG node connection representations

The central focus of this study is the length of motor imagery tasks measured in T-seconds. For each motor imagery task, a collection of 22 EEG signals is employed. Each of the 22 EEG nodes is paired with an associated sensor recording sequence denoted as *r*_*i*_, encompassing measurements such as $r_{i}\in \left[1,N\right]=\left[{\text{ch}}_{1}^{i},{\text{ch}}_{2}^{i},{\text{ch}}_{3}^{i},\ldots ,{\text{ch}}_{m}^{i}\right]\in {\mathbb{R}}^{m}$ via *m* = *T* × *f* time instances.

*r*_*i*_ characterizes the recording sequence for the *i*th EEG node, and [1, *N*] represents the index range for the EEG nodes, where *N* represents the total of 22 nodes. Furthermore, ${\text{ch}}_{t}^{i}$ represents the measurement recorded by the *i*th EEG sensor at the time point *t*, and ℝ^*m*^ indicates that the recording sequence *r*_*i*_ is a vector consisting of real numbers with *m* elements. In the context of the specific details provided, each motor imagery task spans a duration of 4 s (*T* = 4 s), and the EEG signals are sampled at a rate of (*f* = 250) samples per second. Consequently, each EEG signal comprises 1,000 time points, with *m* calculated as the product of *T* and *f*, resulting in *m* = 4 × 250 = 1, 000.

Importantly, these configurations collectively culminate in the formation of the EEG features matrix denoted as *X*_*T*_. This matrix, denoted as $X_{T}=\left[{\text{r}}_{1};{\text{r}}_{2},\ldots ,{\text{r}}_{n}\right]\in {\mathbb{R}}^{n\times m}$, captures the EEG features for the trial *T*. The primary objective of this study is to classify different motor imagery tasks by extracting and utilizing EEG signal features from the matrix *X*_*T*_.

When analyzing EEG nodes, it is important to consider their relationships with neighboring nodes. Due to the constraints of the *X*_*T*_ node dimension, an EEG node can have a maximum of two neighbors. However, in reality, an EEG node typically possesses a multitude of neighboring nodes that capture EEG signals originating from a specific region of the brain. To accurately reflect the spatial relationships between EEG nodes, an undirected spatial graph is created based on the location of the nodes, represented as *G* = (*V, E*) where *V* is the set of vertices/nodes, *V* = *ch*^*i*^|*i* ∈ [1, *N*], containing all the nodes. This graph representation is derived from the adjacency matrix of the EEG nodes, which captures their spatial relationships. This graph representation enhances the capacity of EEG signals to represent distinct brain regions and mitigates the impact of noise by grouping adjacent nodes to represent the central node. This approach enables each EEG node to depend on the support of its neighboring nodes rather than relying solely on its individual measurement. Furthermore, this method enhances the robustness of the EEG data representation in handling missing values.

#### 3.2.2 Adjacency matrix graph (Adj-Graph)

**Figure 3:**
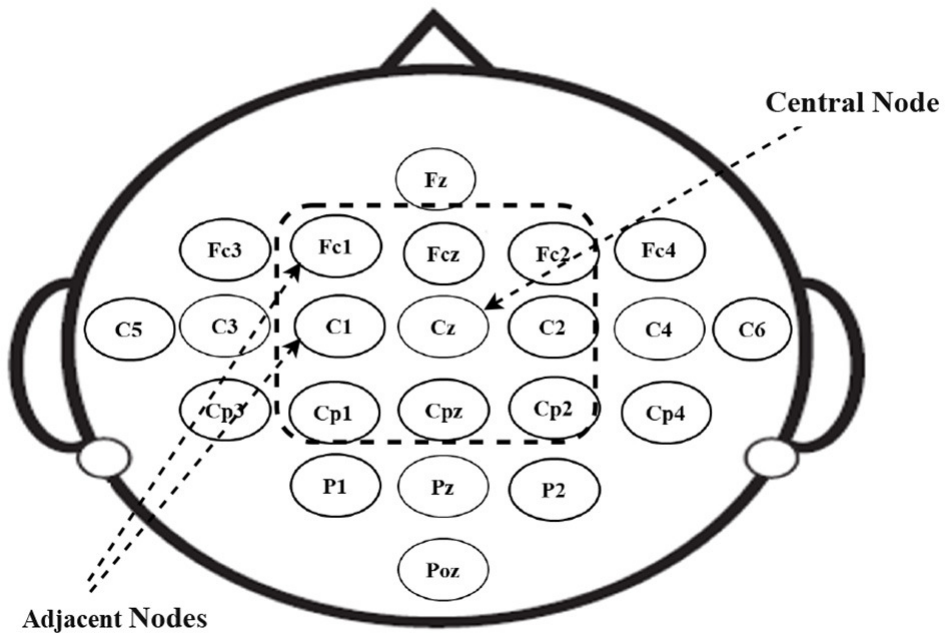
2D channel positions.

The construction of the Adj-Graph is a crucial component of the EEG signal representation in this study. The Adj-Graph is established based on the relationships between EEG nodes and the spatial information captured in the dataset. In the context of the motor imagery tasks, it's essential to represent how different EEG nodes are structurally connected. Figure 3 provides an illustration of the spatial configuration of the 22 EEG nodes. In this configuration, each EEG node is surrounded by neighboring nodes positioned in various directions, including vertically, horizontally, and diagonally. To demonstrate, in Figure 3, node *Cz* has eight neighboring nodes in this configuration (*Cpz, Fcz, C*2, *C*1, *Cp*2, *Cp*1, *Fc*2, *Fc*1). These neighboring nodes are selected based on their spatial proximity to *Cz*, and the process is repeated for all EEG nodes.

To formally represent the structural connections between these EEG nodes, a set of edges *E*_*v*_ is established. This set is defined as $E_{v}=ch^{i}ch^{j}|\left(i,j\right)\in H$. Where *H* includes all pairs of adjacent EEG channels. Each edge in *E*_*v*_ indicates a structural connection between two EEG nodes, reflecting their spatial proximity and relationship within the brain.

It's important to note that every EEG node is considered to be connected to itself as well, as each node should be associated with its own measurement. The adjacency matrix of the Adj-Graph, denoted as *A*_*v*_, represents these structural connections in a square matrix format, with a size of |*V*| × |*V*|, where |*V*| is the number of EEG nodes (22 nodes in the dataset). Each entry in the matrix is a binary value that indicates whether two EEG nodes are structurally connected or not.

Spectral graph theory was used to normalize the adjacency matrix (Kipf and Welling, 2016), as detailed in the following equations:


(1) $$\begin{array}{l} \hat{A_{v}}={\tilde{D_{v}}}^{-\frac{1}{2}}\left(\tilde{A_{v}}\right){\tilde{D_{v}}}^{-\frac{1}{2}} \end{array}$$

where: $\hat{A_{v}}$ represents the normalized adjacency matrix. $\tilde{A_{v}}$ is the modified adjacency matrix obtained by adding the identity matrix *I*_*n*_ to the normalized matrix. $\hat{D_{v}}$ is defined as the diagonal degree matrix of the node and is calculated as follows:


(2) $$\begin{array}{l} \hat{D_{v}}=\text{diag}\left(\underset{j}{\sum }A_{1j},\underset{j}{\sum }A_{2j},\ldots ,\underset{j}{\sum }A_{|v|j}\right) \end{array}$$

${\tilde{D_{v}}}^{-\frac{1}{2}}$ is the inverse square root of the diagonal degree matrix and is calculated as follows:


(3) $$\begin{array}{l} {\tilde{D}}_{v}^{-\frac{1}{2}}=\text{diag}\left(\frac{1}{\sqrt{\sum_{j}A_{1j}}},\frac{1}{\sqrt{\sum_{j}A_{2j}}},\frac{1}{\sqrt{\sum_{j}A_{|v|j}}}\right) \end{array}$$

Details of the adjacency matrix normalization process are provided in Table 1. Therefore, the representation of the Adj-Graph *G*_*v*_ of EEG signals can be expressed as the result of the matrix multiplication between the normalized adjacency matrix of the Adj-Graph and the EEG trial matrix *X*_*T*_: $G_{v}=\hat{A_{v}}\hat{\;}\times X_{T}$.

**Table 1:** Summary of mathematical symbols and explanations.

**Symbol**	**Explanation**
*X*_*T*_	EEG features matrix
*r*_*i*_	Recording sequence for the *i*th EEG node
*N*	Total number of EEG nodes (22 nodes in this dataset)
*m*	Total number of time instances in an EEG signal (1,000 time points in the dataset)
${\text{ch}}_{t}^{i}$	Measurement recorded by the *i*th EEG sensor at time point *t*
ℝ^*m*^	Set of real numbers representing the recording sequence *r*_*i*_
*G*	Undirected spatial graph representing EEG node connections
*V*	Set of vertices/nodes in the graph
*E*	Set of edges in the graph
*E*_*v*_	Set of edges capturing structural connectivity between EEG channels
*A*_*v*_	Adjacency matrix of the Adj-Graph
$\hat{A_{v}}$	Represents the normalized adjacency matrix obtained by applying spectral graph theory to the original adjacency matrix. The hat symbol ˆ indicates that it is a normalized version of *A*_*v*_
$\tilde{A_{v}}$	Represents the modified adjacency matrix obtained by adding the identity matrix *I*_*n*_ to the normalized adjacency matrix $\hat{A_{v}}$. The identity matrix is a square matrix of size *N*×*N*, where (*N* = 22) is the number of nodes, and it has ones on the main diagonal and zeros elsewhere
$\tilde{D_{v}}$	Represents the diagonal degree matrix of the nodes. It is a diagonal matrix of size *V*×*V*, where each diagonal element ${\tilde{D_{v}}}_{i}$ corresponds to the sum of weights (connections) for node *i*. Each diagonal element is calculated as the sum of the weights of node *i* with all its neighboring nodes
${\tilde{D_{v}}}^{-\frac{1}{2}}$	Represents the inverse square root of the diagonal degree matrix, a diagonal matrix of the same size as $\tilde{D_{v}}$, where each diagonal element is the inverse of the square root of the corresponding element in $\tilde{D_{v}}$
${\tilde{D_{v}}}^{-\frac{1}{2}}\left(\tilde{A_{v}}\right){\tilde{D_{v}}}^{-\frac{1}{2}}$	Represents the final step in the normalization process, which involves multiplying the modified adjacency matrix $\tilde{A_{v}}$ by the inverse square root of the diagonal degree matrix on both sides
*G*_*v*_	Representation of the Adj-Graph of EEG signals

#### 3.2.3 Adj-CNNM's configuration

Following the application of the Adj-Graph embedding to EEG signals, a CNN was utilized for encoding and feature extraction in MI data classification. While deep networks exhibit remarkable learning capabilities, it's important to note that delving too deeply into the network layers may not be the most suitable choice for EEG analysis, as highlighted by Schirrmeister et al. (2017).

The configuration of the Adj-CNNM's architecture, as presented in Table 2, includes a 2D convolutional, 2D max pooling, flatten, dropout and dense layers. The 2D convolutional layer was designed with a kernel size of (22, 45) to simultaneously consider all EEG nodes by setting the CNN kernel height to 22, matching the number of EEG nodes in the BCI-IV 2a dataset. The kernel width was empirically extended to 45 to capture a broader range of the input signal, enabling the model to effectively extract relevant features from EEG signals. This adjustment makes it well-suited for the complex MI data classification task. Additionally, 64 CNN filters were experimentally selected to extract informative features across multiple EEG nodes.

**Table 2:** The configurations of Adj-CNNM.

**Layer**	**Kernel size**	**# Kernels**	**Strides**	**Activation**
2D convolutional	(22, 45)	64	(2, 2)	ReLU
2D Max Pool	(1, 50)	1	–	–
Flatten	–	–	–	–
Dropout	0.25	–	–	–
Dense	–	–	–	SoftMax

The use of Rectified Linear Units (ReLU) as the activation function during convolution introduced non-linearity, enhancing feature learning. Spatial resolution was reduced using a stride size of (2, 2). This reduction is advantageous in EEG signal analysis because it helps the model capture essential patterns more efficiently. By focusing on key information and discarding less relevant details, the model becomes more robust in processing EEG signals and extracting relevant features for MI data classification. Further dimensionality reduction was achieved through a 2D max-pooling layer. Consequently, the output of the max pooling layer was flattened into a one-dimensional vector, preparing the data for the subsequent forward propagation through fully connected layers.

Upon testing, it was determined that an optimal balance between preventing overfitting and maintaining model performance was achieved with a 25% dropout rate. The final stage of the model included a dense layer with softmax activation, serving as a classifier to predict the probability distribution of EEG data for four distinct motor imagery task classes.

Adj-CNNM training process was executed with careful consideration of hyperparameters. Adam optimizer, with a learning rate = 0.0001, was chosen due to its efficiency in optimizing complex neural networks. Adam combines the advantages of both the AdaGrad and RMSProp optimizers and adapts learning rates for each parameter individually. This adaptive learning rate scheduling makes it well-suited for training deep neural networks such as the Adj-CNNM. It helps accelerate convergence and enhances the model's ability to escape local minima.

Based on testing, it was determined that training the Adj-CNNM for 1,000 epochs was a deliberate choice to ensure thorough learning and adaptation. EEG data can exhibit subtle patterns that require extended training to be effectively captured. Moreover, this number of epochs was chosen to balance the risk of overfitting and achieve optimal performance while ensuring model convergence.

A batch size of 128 was carefully selected to strike a balance between computational efficiency and model convergence. Larger batch sizes can accelerate training but may lead to suboptimal convergence due to a reduced exploration of the parameter space. A batch size of 128 ensures that a sufficient number of samples are processed in each iteration, facilitating stable and efficient training.

An Early Stopping technique was employed to further enhance the model's training. This mechanism monitors the validation loss during training and halts training when it detects deteriorating performance with a specified patience of 250 epochs. Early Stopping helps prevent overfitting and ensures the model's generalization on unseen data. Further details regarding hyperparameter tuning can be found in Section 4.1.1.

The configuration of the Adj-CNNM was meticulously designed to extract relevant features from EEG signals for MI data classification. The choice of architecture, optimizer, and hyperparameters aimed to strike a balance between model complexity and effectiveness in addressing the challenges of EEG-based MI classification. Evaluations and training of the Adj-CNNM employed various techniques, including Train-Test Split (80/20) and *k*-fold cross-validation, ensuring stability and encompassing all 2,592 trials within the BCI-IV 2a dataset.

Google Colab Pro was the platform of choice for training the Adj-CNNM, harnessing its powerful GPUs, specifically NVIDIA Tesla V100. These GPUs accelerated model training and supported computationally intensive tasks. Additionally, Colab Pro provided high-memory Virtual Machines with 32 GB GPU RAM, enabling efficient processing of large datasets and memory-intensive operations, which were instrumental in achieving efficient and effective model training.

#### 3.2.4 Adj-CNNM limitation

**Figure 4:**
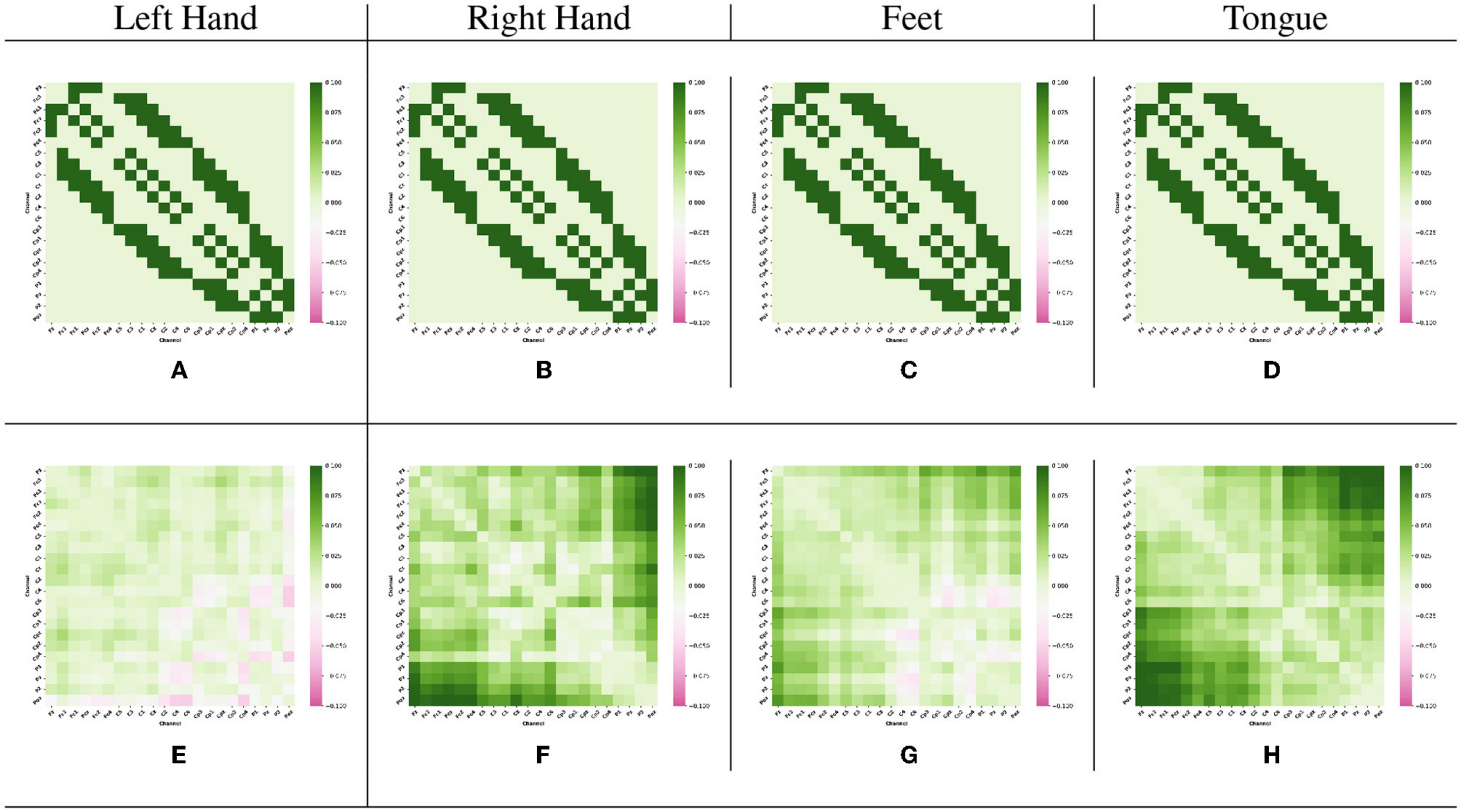
Visualization of Adj-CNNM limitation—illustrating the challenges posed by the Adj-Graph methodology in capturing EEG channel connections across brain regions. **(A)** The average Adj-Graph of trials for subject ONE in Left Hand. **(B)** The average Adj-Graph of trials for subject ONE in Right Hand. **(C)** The average Adj-Graph of trials for subject ONE in Feet. **(D)** The average Adj-Graph of trials for subject ONE in Tongue. **(E)** The average PLV-Graph of trials for subject ONE in Left Hand. **(F)** The average PLV-Graph of trials for subject ONE in Right Hand. **(G)** The average PLV-Graph of trials for subject ONE in Feet. **(H)** The average PLV-Graph of trials for subject ONE in Tongue.

The Adj-Graph methodology offers a robust representation of EEG channel connections and their spatial locations for constructing an EEG graph. However, this approach does have a drawback when it comes to depicting EEG connections across different brain regions. To illustrate this limitation, refer to Figures 4A–D, which presents the adjacency matrix derived from 22 EEG locations forming the Adj-Graph, representing the structural connectivity based on the adjacency matrix.

In certain scenarios, when two channels from distinct brain regions are selected (e.g., channel Fz representing the Frontal lobe and channel Pz representing the Parietal lobe), the adjacency matrix may imply a lack of connection or correlation between these regions. This is because, according to the graph methodology, they are not considered adjacent. Interestingly, empirical testing using the functional connectivity technique, particularly PLV, reveals a significant connection of up to 60% between Fz and Pz, as demonstrated in Figures 4E–H.

These findings emphasize that the graphical representation of the Adj-Graph method may not capture hidden connections across different brain regions. However, this limitation can be effectively overcome by leveraging functional connectivity approaches, enabling a more comprehensive and accurate understanding of the intricate network interactions within the brain. It's important to note that the human brain's functional connectivity doesn't depend solely on spatial proximity; neural synchronization and information transfer can occur between regions that are physically distant but functionally connected. Functional connectivity techniques, such as the Phase Locking Value, tap into the dynamic synchronization patterns of neural oscillations, enabling the detection of underlying relationships that might not be evident in static structural representations.

### 3.3 Graph representation based on functional connectivity

As mentioned earlier, the limitations of the Adj-Graph methodology have led to the exploration of alternative approaches for enhancing the representation of EEG connectivity. To address this limitation, a new methodology based on functional connectivity using PLV is employed.

Functional connectivity captures synchronization and interactions between different brain regions by analyzing the phase relationships of EEG signals. The use of PLV allows the establishment of connections between EEG channels, even when they are not adjacent in terms of spatial location, thereby overcoming the spatial constraints imposed by the Adj-Graph methodology.

**Figure 5:**
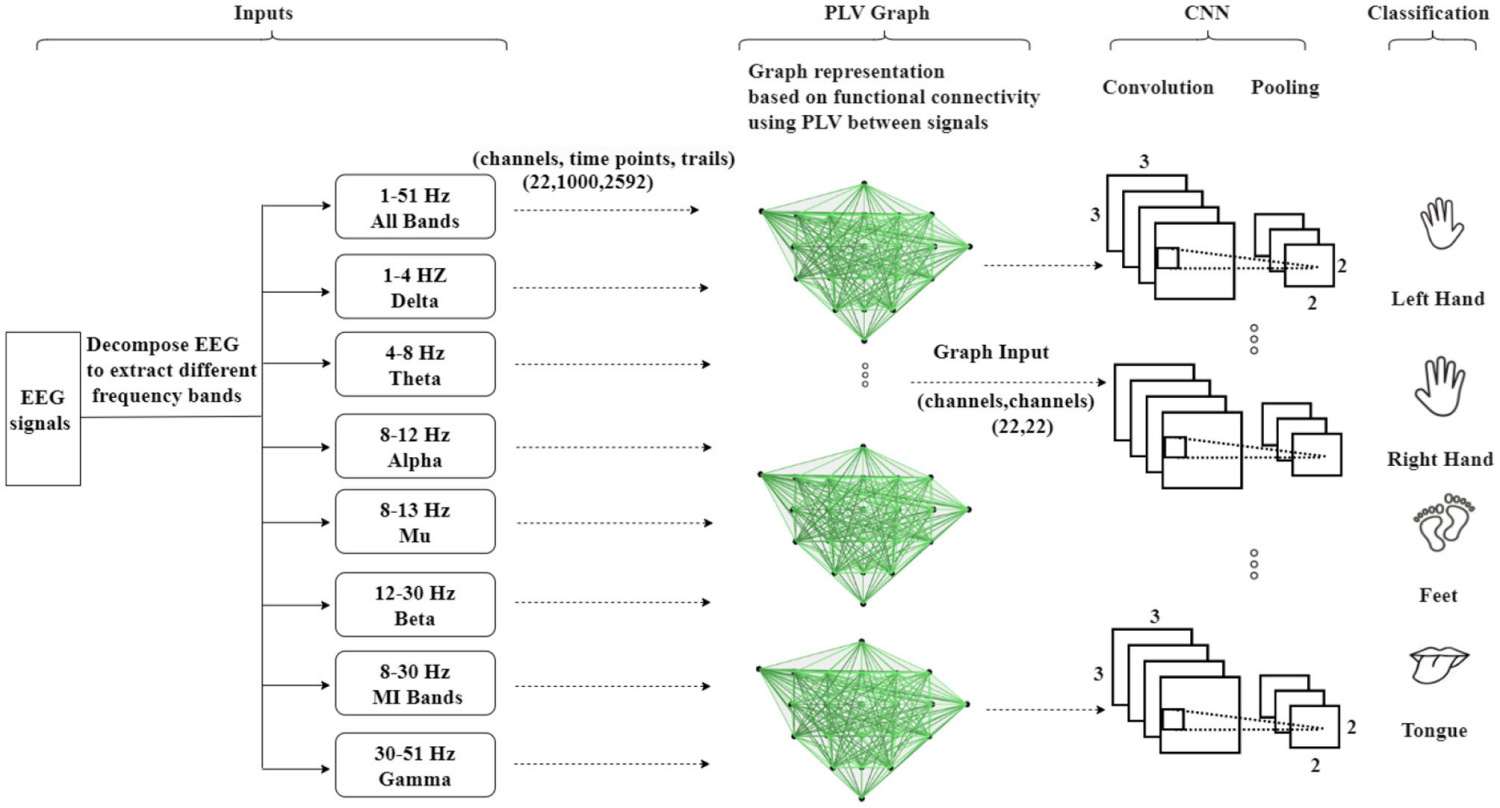
An illustration of the Graph Convolutional Neural Network approach based on functional connectivity (Phase Locking Value—PLV) to classify MI-EEG data, where functional connectivity refers to the statistical associations between different brain regions, indicating the coordinated activity and communication.

As depicted in Figure 5, the second methodology involves embedding the EEG signals into a graph representation using PLV. In this process, PLV between pairs of EEG channels is calculated, creating a connectivity matrix that captures the functional interactions between channels. This matrix is then utilized to construct a functional connectivity graph that represents the dynamic relationships between EEG nodes. The encoded EEG signals are subsequently fed into the neural network model. The framework operates as an end-to-end model, facilitating training through standard back-propagation techniques. The utilization of functional connectivity overcomes the limitation associated with the Adj-Graph methodology, allowing for the capturing of more detailed and comprehensive insights into the complex interactions between EEG channels and brain regions.

#### 3.3.1 EEG channel-connection representations based on PLV

Phase synchronization occurrences are commonly observed in EEG data and have been extensively utilized in studies related to motion imaging and brain-computer interfaces (Piqueira, 2011). When compared to other techniques for assessing the level of phase synchronization between signals, they are preferred. PLV (Goldstein et al., 2008; Gao et al., 2014) is a measure of the synchronization between two EEG channels, indicating the degree to which their signals are correlated in time.

One common approach to represent EEG channel connections based on PLV is by creating a connectivity matrix, also known as a functional connectivity network. In this matrix, each row and column corresponds to a different EEG channel, and the value in each cell represents the PLV between the two corresponding channels. PLV is a measure that indicates the average phase difference between any two signals in terms of their absolute value. This measure allows the phase component of EEG signals to be distinguished from their amplitude component.

The definition formula of PLV is provided in Yi et al. (2014), and can be expressed as follows:


(4) $$\begin{array}{l} PLV\left(t\right)=\frac{1}{N}|\overset{N}{\underset{1}{\sum }}exp\left(i\left(△\varphi_{n}\left(t\right)\right)\right)| \end{array}$$

In this formula, PLV (*t*) is computed at time instant *t* and provides information about the degree of synchronization between the signals. N represents the number of signals being compared, which in the BCI-IV-2a dataset case is 22 signals. Δφ_*n*_(*t*) represents the phase difference between two signals at time *t*, calculated as the difference between their instantaneous phases. The function *exp* represents the exponential function applied to the phase difference Δφ_*n*_(*t*) to convert it into a complex number with both a magnitude and a phase component. $\left|\overset{N}{\underset{1}{\sum }}\right|$ represents the magnitude of the summation of *N* complex numbers. In other words, the equation calculates the sum of the complex numbers obtained from the exponential function and then takes the magnitude of the resulting complex number. The term $\frac{1}{N}$ is the normalization factor used to average the PLV values, ensuring that the PLV values are appropriately scaled considering the number of signals being compared.

Each EEG channel is represented as a node, and the PLV values between the channels are represented as edges. Thus, the graph is created using the PLV connectivity between the different nodes. By constructing a graph using this connectivity, analyzing the patterns of connectivity between different regions of the brain and investigating the functional interactions between them, valuable insights into the network properties of the brain, including information flow dynamics and potential correlations with cognitive functions, are obtained.

#### 3.3.2 PLV graph

As previously mentioned, PLV is a measure of the consistency of the phase difference between two signals. PLV connections between EEG channels can be utilized to construct a graph of EEG connectivity. In this graph, each channel represents a node, and the edges between channels indicate the strength of the functional connectivity between them. The PLV-CNNM graph is constructed based on Equation 4. Building an EEG graph using PLV connections, as shown in Figure 5, involves three key steps:

- Compute the PLV values between each pair of the 22 EEG channels following Equation 4.
- Represent the EEG channels as nodes in the graph, totaling 22 nodes, and establish significant PLV connections as edges between these nodes.
- Assign weights to the edges based on the strength of the PLV values.

In this case, the resulting graph is undirected, meaning that the edges do not have a specific directionality. This approach provides the advantage of identifying patterns of connectivity between EEG channels that are not necessarily directional in nature.

Encoding EEG data into a graph using PLV is a common approach in the analysis of brain connectivity. PLV measures the synchronization between two EEG signals at a specific frequency band. To achieve this, the EEG data undergoes pre-processing and filtering to extract the desired frequency bands, which include 1–51 Hz, δ (1–4 Hz), θ (4–8 Hz), α (8–12 Hz), μ (8–13 Hz), β (12–30 Hz), and 8–30 Hz (the desired frequency band for motor imagery), and γ (30–51 Hz).

Once the EEG data is pre-processed, the next step involves computing the PLV between each pair of EEG channels using Equation 4. PLV values range between 0 and 1, where 0 signifies no synchronization, and 1 indicates perfect synchronization. These connections can be represented as a graph, reflecting the connectivity between different brain regions at varying frequencies. In this graph, each EEG channel serves as a node, and the connections between channels become the edges.

This graph-based method provides insights into the overall network organization of the brain and the patterns of information flow among different regions. Additionally, the PLV graph can be employed to identify network hubs and modules, study changes in network properties, and investigate the relationship between EEG connectivity and other measures of brain function, including cognitive processes such as motor imagery tasks.

#### 3.3.3 PLV CNNM's configuration

Following the encoding of EEG signals using the PLV-Graph, a CNN model was created to better encode and extract time-frequency-domain features for classifying MI tasks, as outlined in Table 3.

**Table 3:** The configurations of PLV-CNNM.

**Layer**	**Kernel size**	**# Kernels**	**Activation**
2D convolutional	(3, 3)	32	Relu
2D Max Pool	(2, 2)	1	–
2D convolutional	(3, 3)	64	–
Dropout	0.25	–	–
Flatten	–	–	–
Dense	–	–	SoftMax

The architecture of the PLV-CNNM consists of two CNN layers with a size of (3, 3) and a single max-pooling layer with a size of (2, 2). When designing these layers, the number of CNN filters was chosen based on extensive testing and evaluation. Specifically, 32 filters were selected for the first layer, and 64 filters for the second layer. This choice stems from the need to capture increasingly complex patterns as the data progresses through the deeper convolutional layers. The decision to use 32 and 64 filters is backed by empirical testing, which revealed that this configuration effectively extracts essential features, especially those related to time-frequency-domain characteristics across multiple EEG nodes. To enhance feature learning, the Rectified Linear Units (ReLU) activation function was employed within the convolutional layers, introducing non-linearity and facilitating the network's ability to recognize intricate patterns and relationships within the EEG data.

To reduce dimensionality and extract essential features, a max-pooling layer was incorporated. Additionally, a dropout regularization layer with a 25% rate was applied to prevent overfitting. The final layer of the PLV-CNNM is a dense layer equipped with a softmax classifier, generating a probability distribution across the four classes. Categorical cross-entropy was employed for error evaluation across all labeled samples. The weights and biases of the network's convolutional layers were trained using batch gradient descent.

During training and evaluation, the PLV-CNNM underwent *k*-fold cross-validation, ensuring a comprehensive assessment across all 2,592 trials in the BCI-IV 2a dataset. The training process took advantage of Google Colab Pro's robust hardware resources, featuring the NVIDIA Tesla V100 GPU and 32 GB of GPU RAM. A batch size of 64 was chosen to ensure efficient and stable training. The training process spanned 800 epochs, which provided ample opportunities for the model to learn and adapt to the data. The Adam optimizer, with a learning rate of 0.001, was employed to efficiently optimize the network's parameters, enhancing convergence and performance. These configurations ensured that the PLV-CNNM was well-equipped to tackle the challenges of EEG-based MI classification. More details about the hyperparameter tuning process for the PLV-CNNM can be found in Section 4.2.1.

## 4 Results

The connectivity of the human brain is a complex phenomenon that involves both structural and functional components. While structural connectivity refers to the anatomical connections of the brain that facilitate communication between different regions, functional connectivity describes the temporal correlation of neural activity between these regions. This results section presents the study's findings on the structural and functional connectivity of the brain. By employing distinct methodologies for analyzing both types of connectivity, the aim is to achieve a more comprehensive understanding of the organization and function of the brain's network.

### 4.1 Adj-CNNM result

#### 4.1.1 Hyperparameter tuning for Adj-CNNM

To optimize the performance of the Adj-CNNM model, a systematic hyperparameter tuning process was undertaken based on nine-fold cross-validation. In the initial phase of experimentation, different filter counts were evaluated while maintaining a fixed kernel size of (22, 45). The results from this stage, as summarized in Table 4, highlighted the impact of filter count variations on the model's performance.

**Table 4:** Hyperparameter tuning results for different filter numbers (Adj-CNNM).

**Filter number**	**Kernel size**	**F1 score (Macro)**	**F1 score (Micro)**	**Accuracy**	**SD**
32	(22, 45)	64.85%	65.27%	65.27%	0.022
**64**	**(22, 45)**	**72.52%**	**72.77%**	**72.77%**	0.045
128	(22, 45)	64.51%	64.81%	64.81%	0.028
256	(22, 45)	64.13%	64.58%	64.58%	0.023

Bold means the best results.

Based on these results, it became evident that the best performance was consistently delivered by a filter count of 64, as indicated by various evaluation metrics, including Macro F1, Micro F1, and Accuracy. Consequently, the subsequent phase of the investigation was directed toward exploring the impact of different kernel sizes while keeping a fixed filter count of 64, as presented in Table 5.

**Table 5:** Hyperparameter tuning results for different Kernels (Adj-CNNM).

**Filter number**	**Kernel size**	**F1 score (Macro)**	**F1 score (Micro)**	**Accuracy**	**SD**
64	(22, 22)	68.48%	68.81%	68.81%	0.018
**64**	**(22, 45)**	**72.52%**	**72.77%**	**72.77%**	0.045
64	(22, 65)	66.39%	66.47%	66.47%	0.025
64	(22, 85)	66.03%	66.28%	66.28%	0.029

Bold means the best results.

This sequential approach allowed the Adj-CNNM model to be fine-tuned effectively, ensuring that the selected configuration was informed by empirical evidence from both filter count and kernel size experiments. Ultimately, this optimization maximized the model's classification capabilities for motor imagery tasks.

#### 4.1.2 Adj-CNNM result

The evaluation of the Adj-CNNM began with an initial assessment using the Train-Test Split method, resulting in promising outcomes: an accuracy of 64.16%. However, for a more comprehensive and rigorous evaluation, the model underwent *k*-fold cross-validation.

The BCI-IV2a dataset encompasses EEG data from nine subjects, with each subject's data collected during a single session comprising six runs. Within each run, 48 trials are distributed across four motor imagery tasks. To ensure that the dataset is partitioned into folds that represent balanced and comprehensive subsets, the goal was to capture the full diversity of trials while maintaining the integrity of the session and subjects. The total number of trials for the first session, serving as the foundation for our cross-validation, amounts to 2,592 (288 trials per session * 9 subjects).

The choice of *k* = 9 aligns with the aim of creating well-distributed and representative folds for cross-validation. In this case, each fold was constructed by mixing data from multiple subjects' first sessions, enabling the assessment of the model's performance across a wide range of trials while preserving the coherence of each fold with the session and subject context.

Furthermore, after systematically testing various values of *k*, it was found that *k* = 9 consistently yielded favorable outcomes in terms of model performance and robustness. Meaningful results were consistently obtained with this configuration, allowing the evaluation of the Adj-CNNM across diverse subsets of data and the minimization of potential impacts from random fluctuations. The choice of *k* = 9 was a deliberate decision based on testing and on the dataset's structure. It enabled the maintenance of session and subject integrity while achieving comprehensive coverage of the dataset, ultimately leading to more reliable and informative model assessments.

Consequently, the Adj-CNNM underwent nine-fold cross-validation across 15 runs, resulting in significant performance improvements compared to the initial train-test split approach. The Adj-CNNM achieved an accuracy of 72.77% with a standard deviation (SD) of 0.045, as shown in Table 7. The utilization of nine-fold cross-validation, with its well-distributed and diverse subsets of data, enabled robust capturing of patterns and generalizations from the EEG trials. The multiple runs further ensured consistent and reliable model performance, minimizing susceptibility to random fluctuations.

### 4.2 PLV-CNNM result

#### 4.2.1 Hyperparameter tuning for PLV-CNNM

To optimize the performance of the PLV-CNNM model, an extensive hyperparameter tuning process based on nine-fold cross-validation was undertaken. Various combinations of filters and kernel sizes were explored to assess their impact on the model's performance. The results of this tuning process, summarized in Table 6, provide valuable insights into the performance of the PLV-CNNM model under different hyperparameter configurations.

**Table 6:** Hyperparameter tuning results for various filters and Kernels (PLV-CNNM).

**Filter combination**	**Kernel size**	**F1 score (Macro)**	**F1 score (Micro)**	**Accuracy**	**SD**
8, 16	(2, 2)	58.40%	58.68%	58.68%	0.036
8, 16	(3, 3)	61.01%	61.23%	61.23%	0.030
32, 64	(2, 2)	66.83%	67.21%	67.21%	0.058
**32, 64**	**(3, 3)**	**74.90%**	**75.10%**	**75.10%**	**0.018**
128, 256	(2, 2)	64.62%	66.47%	66.47%	0.149
128, 256	(3, 3)	70.31%	71.91%	71.91%	0.176
256, 512	(2, 2)	42.05%	47.84%	47.84%	0.235
256, 512	(3, 3)	73.24%	73.50%	73.50%	0.036

Bold means the best results.

Three different 2D Conv filter combinations were tested: (8, 16), (32, 64), (128, 256) along with corresponding kernel sizes, (2, 2) and (3, 3). The influence of these configurations on the model's performance was systematically examined, and the results are presented in the table.

These comprehensive findings enable informed decisions regarding the most suitable hyperparameter configuration for the PLV-CNNM model, considering various evaluation metrics. It is evident that the choice of filters and kernel sizes can significantly impact the model's performance.

#### 4.2.2 PLV-CNNM result

The PLV-CNNM methodology, introduced to address the limitations of the Adj-Graph approach, demonstrates significant advancements in capturing and representing functional connectivity within EEG data. By leveraging PLV as a measure of synchronization between EEG signals, the PLV-CNNM overcomes the spatial constraints posed by the Adj-Graph methodology. This section presents a comprehensive analysis of the PLV-CNNM methodology's outcomes, underscored by its impressive prowess in classifying motor imagery tasks across a diverse range of frequency bands.

The evaluation of the PLV-CNNM was conducted using a robust nine-fold cross-validation technique on the BCI-IV2a dataset, encompassing all 2,592 trials and spanning across 15 runs. By assessing the model's performance across different frequency bands, a comprehensive understanding of its capabilities emerged, as presented in Table 7.

**Table 7:** Performance evaluation for PLV-CNNMs and Adj-CNNM.

**Model**	**F1**	**F1**	**Precision**	**Precision**	**ROC–AUC**	**ROC–AUC**	**Accuracy**	**SD**
	**(Macro)**	**(Micro)**	**(Macro)**	**(Micro)**	**(Macro)**	**(Micro)**		
Adj-CNNM	72.52%	72.77%	73.93%	72.77%	96.00%	95.57%	72.77%	0.045
PLV-CNNM (1–51)	74.90%	75.10%	75.76%	75.10%	96.29%	95.22%	75.10%	0.018
PLV-CNNM (1–4)	93.82%	93.84%	93.88%	93.84%	95.88%	95.85%	93.84%	0.058
PLV-CNNM (4–8)	95.75%	95.72%	95.78%	95.72%	97.60%	97.59%	95.72%	0.044
PLV-CNNM (8–12)	91.87%	91.91%	92.03%	91.91%	98.34%	98.30%	91.91%	0.074
PLV-CNNM (8–13)	90.22%	90.23%	90.26%	90.23%	96.09%	96.05%	90.23%	0.071
PLV-CNNM (8–30)	84.17%	84.30%	84.43%	84.30%	97.51%	97.38%	84.30%	0.030
PLV-CNNM (12–30)	85.69%	85.80%	85.98%	85.80%	96.04%	95.95%	85.80%	0.053
PLV-CNNM (30–51)	86.96%	88.47%	86.71%	88.47%	97.73%	97.76%	88.47%	0.074

Table 7 offers a comprehensive overview of the PLV-CNNM's performance, depicting its accuracy and corresponding standard deviation for various frequency bands. Notably, the PLV-CNNM showcases remarkable accuracy in classifying motor imagery tasks across distinct frequency ranges. Specifically, the θ frequency band stands out with a remarkable accuracy of 95.72% achieved by the PLV-CNNM, which can be attributed to its adeptness in capturing neural synchronization patterns closely linked to cognitive functions essential for motor imagery tasks. Specifically, the θ frequency band is prominently associated with memory formation and attentional processes, both of which play pivotal roles in motor imagery. As participants engage in mentally stimulating movements, subjects rely on memory recall to reproduce specific actions. The PLV-CNNM's exceptional accuracy in this frequency band can thus be explained by its proficiency in detecting and leveraging the nuanced neural synchronization patterns indicative of memory encoding and attentional focus. Additionally, the PLV-CNNM's success aligns with empirical observations of increased theta band activity during motor imagery (Erfani and Erfanian, 2004; Cruikshank et al., 2012).

Additionally, the δ, α, μ, β, 8–30 Hz—desired frequency band for MI, γ, and All bands (1–51 Hz) exhibit favorable accuracy values of 93.84%, 91.90%, 90.22%, 85.80%, 84.30%, 88.47%, and 75.10%, respectively, further reinforcing the model's proficiency in accurately classifying motor imagery tasks.

### 4.3 Result comparison between Adj-CNNM and PLV-CNNM

The comparison between the Adj-CNNM and PLV-CNNM unveils distinct performance characteristics in the classification of motor imagery tasks. Both models demonstrate significant advancements in capturing and representing connectivity patterns within EEG data, albeit through different approaches. The evaluation matrix, presented in Table 7, showcases detailed performance metrics for both models, offering a comprehensive view of their capabilities across various frequency bands.

Adj-CNNM: The Adj-CNNM achieved a superior classification accuracy of 72.77%, showcasing its remarkable ability to classify motor imagery tasks. This exceptional accuracy can be attributed to its unique utilization of a graph-based representation of EEG signals. The incorporation of adjacency matrices to encode intricate spatial interconnections between brain regions enables the model to learn more informed and relevant features, contributing to its outstanding performance in motor imagery classification.

PLV-CNNM: The PLV-CNNM effectively addresses limitations in capturing connections between brain regions through the use of functional connectivity approaches, such as PLV. This adaptation led to remarkable accuracy in the resulting PLV-CNNM models, reaching up to 95.72% in specific frequency bands. Notably, the PLV-CNNM model showcases remarkable accuracy in classifying motor imagery tasks across distinct frequency ranges, including α, μ, β, and γ, reinforcing its proficiency in accurately classifying motor imagery tasks. The low SD values accompanying each accuracy result underscore the consistent and reliable performance of the model across different frequency bands, highlighting its capacity to accommodate the inherent variability present in EEG trials.

While the Adj-CNNM excels in graph-based representation and captures complex spatial interconnections, the PLV-CNNM, leveraging its unique approach based on PLV and a diverse range of frequency bands, demonstrates superior accuracy, F1-scores, precision, and ROC-AUC scores. These results underscore the PLV-CNNM's prowess in capturing the nuances of neural synchronization patterns and functional connectivity within EEG data, making it a valuable candidate for motor imagery classification tasks. Nevertheless, both the Adj-CNNM and PLV-CNNM offer valuable contributions to motor imagery classification.

## 5 Discussion

### 5.1 Performance

#### 5.1.1 Adj-CNNM performance

For this study, the BCI-IV2a dataset was chosen due to its even distribution, enabling a comprehensive evaluation of the performance of the proposed Adj-CNNM. The performance assessment considered a range of evaluation metrics. These metrics included classification accuracy, F1-score (both Macro and Micro), precision (both Macro and Micro), ROC-AUC (both Macro and Micro), and SD. Comparative results for these metrics are presented in Table 7.

To ensure an unbiased comparison and highlight the superiority of the proposed approach, state-of-the-art methods were chosen as benchmarks, as shown in Table 8. Initially, Adj-CNNM was compared to EEGNet (Lawhern et al., 2018), which combines various EEG feature extraction techniques to create a unified approach for handling diverse BCI scenarios. Subsequent comparisons were made with CroppedTrainingCNN (CTCNN) (Schirrmeister et al., 2017), which explored various CNN architectures and introduced a superior cropped training technique compared to the conventional trial-based training method.

**Table 8:** State-of-the-art and baseline models comparison with proposed methods.

**Comparison model**	**Accuracy**	**SD**
EEGNet (Lawhern et al., 2018)	51.31%	0.051
CTCNN (Schirrmeister et al., 2017)	47.67%	0.150
EEG image (Bashivan et al., 2015)	32.47%	0.043
Cascade model (Zhang et al., 2019b)	31.83%	0.039
Parallel model (Zhang et al., 2019b)	32.67%	0.449
FBCSP (Ang et al., 2008)	35.69%	0.083
PSD-SVM (Oikonomou et al., 2017)	36.11%	0.081
CRAM (Zhang et al., 2019a)	59.10%	0.108
NG-RAM (Zhang et al., 2020)	60.11%	0.099
DG-RAM (Zhang et al., 2020)	59.64%	0.097
SG-RAM (Zhang et al., 2020)	59.00%	0.101
T-WaveNet-Haar (Minhao et al., 2021)	43.12%	NA
T-WaveNet (without feature fusion) (Minhao et al., 2021)	61.03%	NA
T-WaveNet (Minhao et al., 2021)	63.01%	NA
Multi-branch 3D CNN (Zhao et al., 2019)	75.02%	7.344
Global spatial convolution (Liao et al., 2020)	74.60%	NA
Local spatial convolution (Liao et al., 2020)	71.80%	NA
Parallel spatial convolution (Liao et al., 2020)	73.20%	NA
Attention based inception model (Amin et al., 2021)	74.70%	NA
Incep-EEGNet (Riyad et al., 2020)	74.07%	NA
MI-EEGNET (Riyad et al., 2021)	74.61%	NA
Multi-domain information fusion (Wang et al., 2023)	75.00%	NA
CNN (without Adj-Graph)	32.63%	0.033
CNN (without PLV-Graph)	40.28%	0.027
Adj-CNNM	72.77%	0.045
PLV-CNNM [All bands (1–51 Hz)]	**75.10%**	0.018
PLV-CNNM (1–4 Hz—δ band)	**93.84%**	0.058
PLV-CNNM (4–8 Hz—θ band)	**95.72%**	0.044
PLV-CNNM (8–12 Hz—α band)	**91.90%**	0.074
PLV-CNNM (8–13 Hz—μ band)	**90.22%**	0.071
PLV-CNNM (12–30 Hz—β band)	**85.80%**	0.053
PLV-CNNM (8–30 Hz—desired frequency band for MI)	**84.30%**	0.030
PLV-CNNM (30–51 Hz—γ band)	**88.47%**	0.074

The EEG-Image method (Bashivan et al., 2015) was also compared, which uses spectral, spatial, and temporal characteristics exhibited by EEG signals and a convolutional recurrent model to classify mental workloads. The Cascade and Parallel models (Zhang et al., 2019b), which retain EEG spatial information by taking into account adjacent EEG nodes, were also evaluated.

The evaluation also included classic EEG analysis techniques. These techniques encompassed PSD-SVM (Oikonomou et al., 2017), known for generating time-frequency-based features frequently used in MI-EEG analysis, and FBCSP (Ang et al., 2008), a popular classic approach. State-of-the-art methods, as well as two baseline CNN models, were also included. Additional details are available in the ablation study in Section 5.3.

Importantly, the efficacy of the graph-based representation of EEG signals in the Adj-CNNM was demonstrated through a comparison with the CRAM model (Zhang et al., 2019a) and the Graph-based Convolutional Recurrent Model (NG-RAM) (Zhang et al., 2020), which included three different architectures. The study also compared the proposed method to T-WaveNet (Minhao et al., 2021), which used power spectral analysis to decompose input signals into multiple frequency sub-bands for sensor data analysis using a novel tree-structured wavelet neural network (T-WaveNet).

Notably, Adj-CNNM, the proposed model, was also compared to the groundbreaking study by Zhao et al. (2019), which introduced a 3D representation of EEG data for MI classification. This innovative approach preserved both spatial and temporal information, harnessing the power of a multi-branch 3D CNN to extract MI-related features. The fusion of this 3D EEG representation with the multi-branch 3D CNN yielded remarkable results.

In addition to the aforementioned methods, Adj-CNNM was compared to several other recent studies in the field of MI classification. Liao et al. (2020) explored the “EEG as image” paradigm for MI classification, introducing three distinct deep learning models that employ varying spatial convolution strategies. Their global model achieved promising classification accuracy, demonstrating the profound impact of spatial filters on classification accuracy. Amin et al. (2021) harnessed the power of deep learning with an attention-based CNN model. Their Multi-CNN Feature Fusion approach addressed inter-subject and inter-session variability, achieving superior performance compared to existing methods.

The results, as shown in Table 8, indicate that Adj-CNNM not only outperforms many methods but also achieves competitive results. Its primary strength lies in its graph embedding representation, a feature that enhances its performance when compared to deep learning models. Unlike pure deep learning models such as EEGNet and CTCNN, which lack specific data representations, the graph representation of the Adj-CNNM includes and encodes the spatial interconnections among EEG nodes. This enables more effective neural network analysis of EEG signals. In contrast to the spatial representation used in the EEG-Image method, the graph representation technique of Adj-CNNM doesn't require data implantation, thereby avoiding potential noise. Additionally, unlike the approach in Zhang et al. (2019b), the Adj-CNNM utilizes an adjacency matrix to improve the efficiency of EEG data encoding.

#### 5.1.2 PLV-CNNM performance

PLV, a metric indicating the degree of phase consistency between two signals, is used in EEG analysis to measure functional connectivity among various brain regions. This information is represented as a graph, with each node representing a specific brain region and the edges reflecting the strength of functional connectivity between those regions. This approach offers a visual representation of the intricate network of connections between different brain regions, aiding in the comprehension of brain activity coordination and the collaboration of various regions to support cognitive functions.

Figures 4E–H present PLV correlation matrices for subject one within the 8–30 Hz frequency band, which is essential for MI tasks involving the left hand, right hand, feet, and tongue. The deliberate selection of subject one was motivated by the aim to gain insights into the functional connectivity patterns during motor imagery tasks. This choice allows for an exploration of how subject one exhibits variations in connectivity and coordination between brain regions across the MI tasks. The 8–30 Hz frequency range includes the α, μ, and β rhythms, which play critical roles in motor planning, preparation, and cognitive processes. Relaxed wakefulness and sensory inhibition are signified by the α rhythm (8–12 Hz). The μ rhythm (8-13 Hz), known as the sensorimotor rhythm, is closely linked to motor-related activities, reflecting involvement in motor planning and execution. Active cognitive engagement, motor execution, and sensorimotor integration are associated with the β rhythm (12–30 Hz).

As motor imagery tasks are performed by subject one, the PLV correlation matrix unveils the complex interactions between electrodes. Significantly higher connectivity is observed in certain electrodes, indicating potential variations in neural network dynamics and activation strategies. This suggests that different contributions to motor imagery may exist among specific brain regions across various tasks.

Figures 4E–H reveal intriguing variations in phase synchronization patterns observed during motor imagery tasks in subject one, particularly in the 8–30 Hz frequency range. Distinct PLV and functional connectivity features are observed among brain regions in certain task categories, highlighting the non-uniform nature of neural processes during motor imagery. These findings suggest varying levels of phase synchronization and connectivity among brain regions related to these tasks, highlighting the complexity of neural coordination during motor imagery.

The model's performance was evaluated using different evaluation matrices, including accuracy, F1-score (both Macro and Micro), precision (both Macro and Micro), ROC-AUC (both Macro and Micro), and SD, based on a nine-fold cross-validation procedure, as presented in Table 7.

An accuracy of 75.10% was achieved by the PLV-CNNM when considering all frequency bands combined (1–51 Hz) with a standard deviation of 0.018. However, the crucial frequency band for motor imagery classification is (8–30 Hz), which encompasses α (8–12 Hz), μ (8–13 Hz), and β (12–30 Hz) waves. The PLV-CNNM was initially evaluated for the desired frequency band separately, with accuracies of 91.90%, 90.20%, and 85.80% achieved, with standard deviations of 0.074, 0.071, and 0.053 for α, μ, and β, respectively. Additionally, the PLV-CNNM was evaluated for the combined (8–30 Hz) band, with an accuracy of 84.30% achieved and a standard deviation of 0.03. These results indicate a relatively consistent performance across most frequency bands, with standard deviations ranging from 0.044 to 0.075.

When compared to other notable studies in the domain of MI classification, PLV-CNNM exhibits compelling performance, as shown in Table 8. In a study by Zhao et al. (2019), a pioneering 3D EEG representation achieved an accuracy of 75.015%. Similarly, in the work of Liao et al. (2020), the “EEG-as-image” approach yielded a remarkable classification accuracy of 74.60%, underscoring the impact of spatial filters. On the other hand, Alwasiti et al. (2020) employed deep metric learning to address inter-individual variability and showcased promising results, achieving convergence with minimal training samples. Amin et al. (2021) introduced an attention-based Inception model, achieving an accuracy of 74.70% and effectively addressing inter-subject and inter-session variability. Additionally, studies like Riyad et al. (2020) and Riyad et al. (2021) leveraged architectures based on Inception, achieving accuracies of 74.07 and 74.61%, respectively. Furthermore, in the recent study by Wang et al. (2023), the authors tackled low classification accuracy and EEG channel selection challenges. Their innovative approach led to an accuracy of 75.00%.

These studies present innovative approaches to MI classification, yet PLV-CNNM's distinctive focus on functional connectivity through PLV and specific frequency band analysis sets it apart, contributing to its competitive performance and enhanced understanding of neural coordination during motor imagery tasks.

### 5.2 Interpretations

#### 5.2.1 Adj-CNNM interpretations

**Figure 6:**
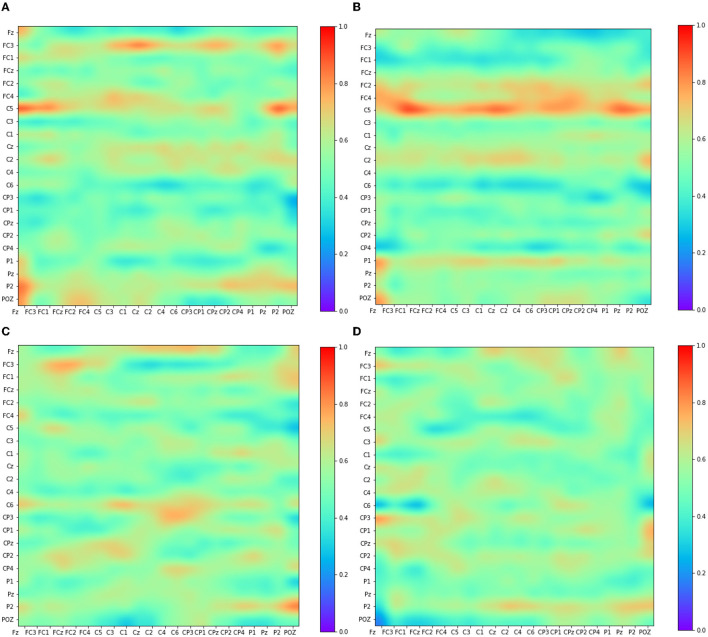
Visualization of some convolutional feature map representations. The depicted feature maps were strategically chosen due to their focus on key EEG nodes (C3, Cz, and C4) known for conveying significant motor-related information. **(A)** Kernel 6. **(B)** Kernel 7. **(C)** Kernel 8. **(D)** Kernel 9.

This section explores the EEG feature learning process in Adj-CNNM, providing valuable insights into the classification of MI data. Figure 6 visually presents the convolutional feature maps generated during this intricate process. The selection of these feature maps deliberately targets distinct EEG nodes, including the prominent C3, Cz, and C4 channels, which have previously been established as carriers of the most discriminative MI-EEG information (Oikonomou et al., 2017). This strategic focus on essential EEG nodes underscores the importance of the primary motor cortex and the identified EEG channels in Adj-CNNM's learning process, enabling it to effectively capture the crucial EEG features required for successful MI classification.

The primary motor cortex plays a pivotal role in classifying motor imagery tasks. Within the widely-used 10–20 EEG system placement, the channels C3, C4, and Cz have been identified as the most informative channels for capturing and classifying motor-related brain activity. Figure 6 illustrates Adj-CNNM's learning process, demonstrating how EEG features are acquired for MI data classification. Similar to the findings in Zhang P. et al. (2018), Adj-CNNM's CNN layers specifically target intricate brain regions of relatively smaller scale, facilitating the extraction of essential EEG features. Moreover, Adj-CNNM's CNN layers emphasize the frontal and central (FC and C) regions of the brain, in line with existing literature (Shin et al., 2018). The kernels (kernel 6, 7, 8, and 9) are distinctly focused on C3, Cz, and C4, reaffirming the significance of these key channels. This alignment with vital channels not only showcases the kernels' capacity to discern and extract vital features from channels that prominently convey motor-related information but also validates Adj-CNNM's effectiveness in efficiently targeting crucial regions for motor imagery interpretation. Furthermore, Adj-CNNM's adaptability in its CNN layer enables expansion to encompass other EEG nodes, thereby enhancing its ability to distinguish between various types of motor imagery.

The intricate process of EEG feature learning in Adj-CNNM, guided by the precise targeting of essential EEG nodes and a focus on key channels, underscores its effectiveness in capturing vital features for successful motor imagery classification. This contributes to the understanding of brain activity patterns and enhances the potential for diverse applications in neuroimaging research.

#### 5.2.2 PLV-CNNM interpretations

**Figure 7:**
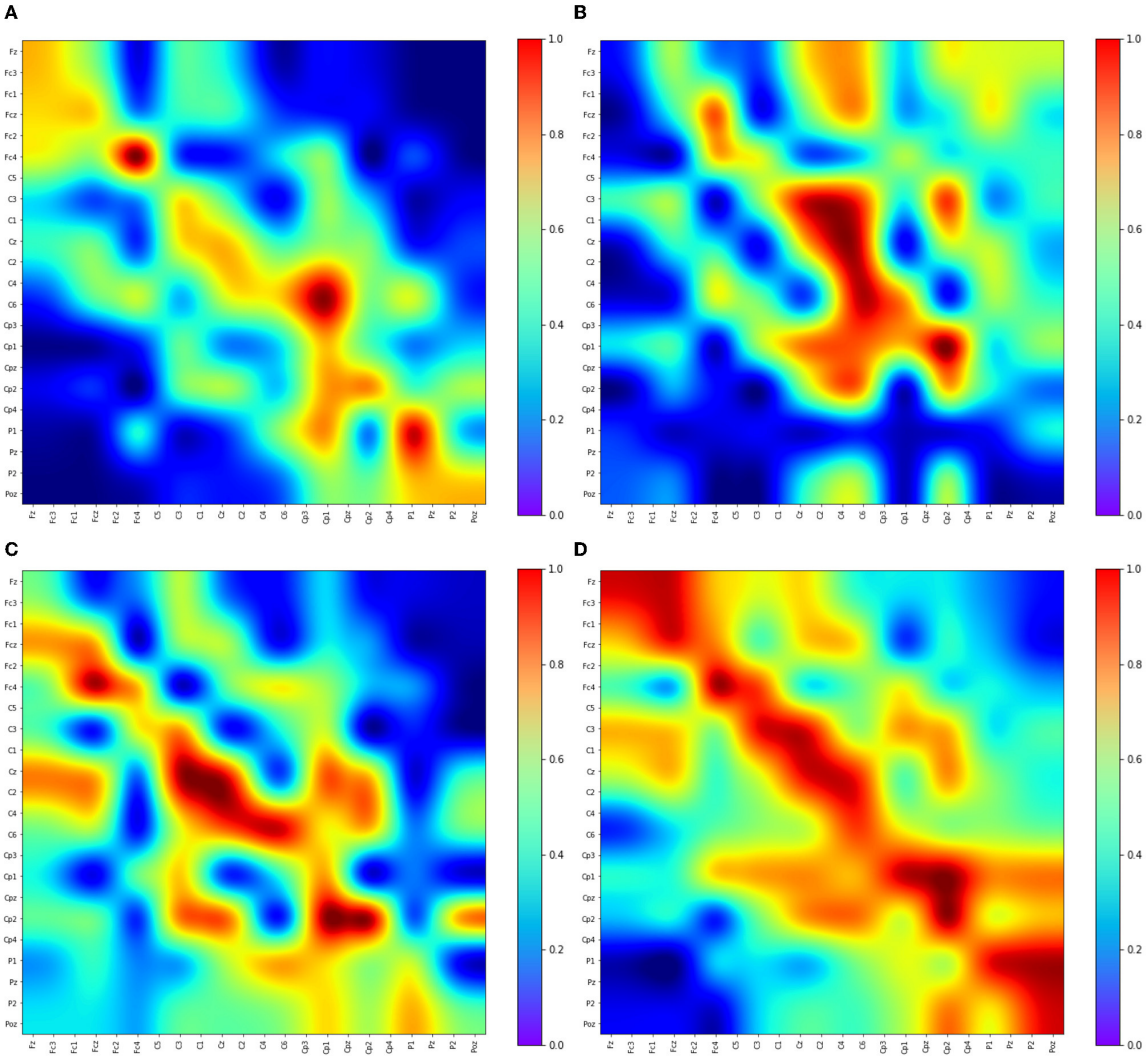
Visualizing Convolutional Feature Maps (Kernels) in the 8–30 Hz Frequency Band: Emphasizing Frontal, Central, and Parietal Brain Regions and Their Interconnections During Motor Imagery. This figure showcases selected feature maps (kernels) from the PLV-CNNM through channels from the frontal cortex (Fz, Fcz, Fc3, Fc1, Fc2, Fc4), central cortex (C5, C3, C1, Cz, C2, C4, C6, Cp3, Cp1, Cp2, Cp4, Cpz), the parietal cortex (P1, P2, Pz, and Poz). These maps not only illuminate the significance of these brain regions in motor imagery interpretation but also reveal their interconnections, providing a comprehensive view of neural dynamics during motor imagery. **(A)** Kernel 23. **(B)** Kernel 8. **(C)** Kernel 11. **(D)** Kernel 4.

This section delves into the intricate process through which PLV-CNNM acquires essential EEG features for MI data classification. Figure 7 visually depicts the learning dynamics within the CNN layers.

- Emphasis on distinct brain regions: Building upon insights from previous studies (Shin et al., 2018; Zhang P. et al., 2018), which highlighted the proficiency of CNN layers in targeting specific brain regions for effective feature extraction, the PLV-CNNM demonstrates a similar emphasis on distinctive brain areas. Notably, the frontal cortex (FC), the central region (C), and the parietal region (P) take center stage in its feature acquisition process, aligning with the significance of these regions in cognitive tasks (Shin et al., 2018) underscores the importance of these regions in cognitive tasks.
- Frontal region activities: Within the 8–30 Hz band, Figure 7A reveals pronounced activities in the frontal region. Channels such as Fz, Fc3, Fc1, Fcz, Fc2, and Fc4, located in the frontal cortex, exhibit discernible levels of activation. These observations provide valuable insights into the functional dynamics of the frontal region during motor imagery tasks.
- Central region activities: In the same frequency band, Figures 7B, C illustrate activities within the central region. Channels like C5, C3, C1, Cz, C2, C4, C6, Cp3, Cp1, Cp2, Cp4, and Cpz, central to the motor imagery process, demonstrate significant activity levels. Of particular note is the activation of multiple feature maps at the C3, Cz, and C4 EEG nodes, which serve as vital sources of information for motor imagery EEG classification (Oikonomou et al., 2017). Figure 7 provides a visual representation of how the kernels within the CNN layers purposefully engage with distinct channels, with a particular focus on C3, Cz, and C4. This representation underscores the unique contributions of these nodes to the intricate process of EEG feature extraction and subsequent classification, enhancing our understanding of the interplay between neural dynamics and cognitive task performance.
- Interregional connectivity: Figure 7C offers another feature map overview of activities in various brain regions, with a specialized focus on the frontal, central, and parietal brain regions during the desired frequency band for motor imagery classification (8–30 Hz). This visualization provides insights into the patterns and distribution of brain activity associated with the specific task, enhancing the understanding of the underlying neural dynamics. Moreover, This visualization reveals vital information from distinct brain regions: the frontal cortex (Fz, Fcz, Fc3, Fc1, Fc2, and Fc4), central cortex (C5, C3, C1, Cz, C2, C4, C6, Cp3, Cp1, Cpz, Cp2, and Cp4). These distinctions provide a detailed view of connectivity and interactions within and between these critical brain regions during motor imagery tasks. Additionally, the PLV-CNNM effectively demonstrates robust connections between the frontal and central cortex via channels spanning from Fz to C6. Activities in the central cortex are evident through channels Cp3 to Cp4, while functional connections link the parietal cortex via channels P1, Pz, and Poz.
- Consistent activation patterns across tasks: Figure 7D reveals that during motor imagery tasks involving different body parts, specific brain regions may exhibit varying activity levels. However, several studies have identified consistent activation patterns across various tasks. For instance, motor imagery tasks associated with hand movements often trigger activation in areas of the contralateral primary motor cortex (M1) and premotor cortex (PMC) specific to the imagined side of the body (Bai and Fong, 2020). Visualizing movements of the right hand would activate the left M1 and PMC, while imagining movements of the left hand would trigger activation in the right M1 and PMC. The most commonly utilized channels to measure activity in M1 and PMC are C3, C4, Fc3, and Fc4 (Hoshino et al., 2020). Figure 7D depicts the activities within the M1 and PMC areas for the C3, C4, Fc3, and Fc4 channels across the 8–30 Hz frequency bands.
- Distinct activation patterns in different tasks: Similarly, motor imagery tasks involving foot movements activate areas in the contralateral M1 and PMC, with slightly different locations of activation compared to hand imagery tasks (Spedden et al., 2020). Motor imagery tasks that involve tongue movements activate the Supplementary Motor Area (SMA), a region involved in movement planning and coordination, represented by channels Fz and Cz, as depicted in Figure 7D.

The effective demonstration of the PLV-CNNM reveals notable connections between distinct brain regions during motor imagery tasks, particularly within the 8–30 Hz frequency range. This frequency band holds significance for motor imagery classification, highlighting its relevance in understanding neural processes associated with these tasks.

### 5.3 Ablation study

An ablation study was conducted to assess the impact of the graph-based representations on the performance of the motor imagery classification models. Specifically, the influence of the adjacency matrix-based graph representation (Adj-Graph) and the Phase Locking Value-based graph representation (PLV-Graph) on the performance and robustness of the classification models was investigated.

CNN (without Adj-Graph): In this configuration, the use of the adjacency matrix-based graph representation was excluded, resulting in a baseline CNN architecture applied directly to EEG signals without incorporating spatial interconnections between brain regions.

CNN (without PLV-Graph): In this model configuration, the PLV-Graph was omitted from the pipeline, and the CNN architecture was applied directly to EEG signals without considering functional connectivity features derived from the Phase Locking Value.

Results and analysis: CNN (without Adj-Graph): The configuration that omitted the adjacency matrix-based graph representation achieved an accuracy of 32.64% with a standard deviation of 0.033, as shown in Table 9. This result underscored the substantial impact of the Adj-Graph on the model's performance. The exclusion of spatial interconnections between brain regions led to a significant drop in classification accuracy.

**Table 9:** Ablation experiment to assess the graph representation impact on the model's performance.

**Model**	**F1**	**F1**	**Precision**	**Precision**	**RO–AUC**	**ROC–AUC**	**Accuracy**	**SD**
	**(Macro)**	**(Micro)**	**(Macro)**	**(Micro)**	**(Macro)**	**(Micro)**		
CNN (without Adj-Graph)	28.34%	32.64%	32.01%	32.64%	56.18%	55.70%	32.64%	0.033
CNN (without PLV-Graph)	40.52%	40.28%	40.64%	40.28%	65.12%	65.54%	40.28%	0.027
Adj-CNNM	72.52%	72.77%	73.93%	72.77%	96.00%	95.57%	72.77%	0.045
PLV-CNNM	74.90%	75.10%	75.76%	75.10%	96.29%	95.22%	75.10%	0.018

CNN (without PLV-Graph): When the PLV-Graph was not incorporated, an accuracy of 40.28% was achieved with a standard deviation of 0.027. While this model outperformed the configuration without the Adj-Graph, the value of PLV-Graph in enhancing the model's performance was clearly demonstrated. The removal of functional connectivity features derived from the Phase Locking Value led to a reduction in classification accuracy.

Interpretation: The critical roles played by the Adj-Graph and PLV-Graph in the classification of motor imagery tasks were revealed by the results of the ablation study. The absence of the Adj-Graph substantially impacted the model's ability to capture complex spatial interconnections, leading to a significant decrease in accuracy. Similarly, the removal of the PLV-Graph resulted in a noticeable reduction in classification accuracy, indicating the importance of functional connectivity features in capturing nuanced neural synchronization patterns. The complementary nature of the two graph-based representations was underscored, where the Adj-Graph enhanced the spatial representation of EEG data, while the PLV-Graph captured the temporal dynamics and functional connectivity within the neural network. The successful combination of these graph representations in the Adj-CNNM and PLV-CNNM models led to significant improvements in motor imagery classification accuracy and robustness. Overall, the value of these graph-based representations in modeling the complex relationships within EEG data and their critical roles in enhancing the performance of motor imagery classification models was demonstrated by the ablation study.

## 6 Conclusion

In this study, a proposed approach to EEG motor imagery classification has been introduced, harnessing the power of deep learning and graph embedding techniques that utilize brain connectivity to enrich the understanding of brain function. The focal point of this work lies in the development of two distinct graph-based convolutional neural networks: the Adj-CNNM and the PLV-CNNM. The Adj-CNNM is characterized by the utilization of structural brain connectivity to embed spatial information and has demonstrated remarkable performance, achieving an accuracy of 72.77%. Notably, this approach distinguishes itself from conventional spatial filtering methods by achieving independence from individual and task-specific dependencies, offering a broader comprehension of brain network organization.

To transcend the limitation of structural connectivity, the PLV-CNNM was introduced, which integrates functional connectivity patterns. Achieving an overall accuracy of 75.10% across the 1–51 Hz frequency range and exceptional individual accuracies of 91.9%, 90.2%, 85.8% and 84.30% for the critical frequency band including α, μ, β and 8–30 Hz—desired frequency band for MI waves, the PLV-CNNM successfully uncovers robust connections between various brain regions during motor imagery tasks. Notably, prominent interconnections emerge between the frontal and central cortex and the central and parietal cortex. These findings significantly contribute to expanding the understanding of brain connectivity patterns and their pivotal roles in cognitive processes.

In addition to the development of the Adj-CNNM and PLV-CNNM models, an ablation study was conducted to assess the impact of the graph-based representations on the performance of the motor imagery classification models. The influence of the adjacency matrix-based graph representation (Adj-Graph) and the Phase Locking Value-based graph representation (PLV-Graph) on the performance and robustness of the classification models was investigated. It was found that the Adj-Graph substantially impacted the model's ability to capture complex spatial interconnections, resulting in a significant decrease in accuracy. Similarly, the removal of the PLV-Graph led to a noticeable reduction in classification accuracy, indicating the importance of functional connectivity features in capturing nuanced neural synchronization patterns. These findings emphasize the critical roles played by the Adj-Graph and PLV-Graph in the classification of motor imagery tasks.

The presentation of a comprehensive comparative analysis of diverse brain connectivity modeling methods not only showcases the effectiveness of the proposed models but also provides a valuable resource for researchers seeking to explore brain connectivity from different perspectives. In conclusion, this study exemplifies the potential of graph embedding and deep learning to untangle the complexities of brain connectivity. The advancements achieved in EEG motor imagery classification through the Adj-CNNM and PLV-CNNM models hold the promise of deepening the understanding of neurological conditions and cognitive processes.

## 7 Future work

In this study, the introduction of deep learning and graph embedding techniques for EEG motor imagery classification has laid the foundation for promising future research directions. Firstly, cross-dataset validation can be pursued to assess the generalizability and robustness of the proposed models across diverse EEG datasets. Further hyperparameter tuning and experimentation with various model architectures and optimization strategies may yield improved classification accuracy.

The inclusion of temporal information, possibly through the exploration of RNNs or attention mechanisms, can enhance the models' ability to capture temporal dependencies in EEG data. Expanding the scope to encompass different cognitive tasks beyond motor imagery classification would broaden the applicability of these models. Furthermore, exploring other types of brain connectivity, such as effective connectivity, can contribute to a more comprehensive understanding of brain function and connectivity patterns.

Moreover, further exploration of biomedical applications, including early diagnosis or monitoring of neurological conditions, as well as applications in neurorehabilitation and assistive technology, could significantly impact healthcare. The development of techniques to enhance model interpretability would provide valuable insights into brain connectivity patterns, making the models more accessible and informative for researchers and clinicians.

Lastly, the integration of EEG data with other neuroimaging modalities, like fMRI or MEG, offers a comprehensive view of brain connectivity and function, potentially leading to more robust and nuanced insights. Open-sourcing the code and models developed in this study would promote collaboration and facilitate further research in the field, enabling the scientific community to build upon and refine the proposed methods for EEG-based brain connectivity analysis.

## Data availability statement

The datasets presented in this study can be found in online repositories. The names of the repository/repositories and accession number(s) can be found in the article/Supplementary material.

## Author contributions

AA: Formal analysis, Investigation, Methodology, Software, Validation, Visualization, Writing—original draft, Writing—review & editing. Y-KW: Scope identifying, Writing—review & editing, Supervision.
