# Supplementary figures and images for: Revealing brain connectivity: graph embeddings for EEG representation learning and comparative analysis of structural and functional connectivity
Source: Front Neurosci. 2024 Jan 8;17:1288433. doi: 10.3389/fnins.2023.1288433 (PMC10804888; doi:10.3389/fnins.2023.1288433)

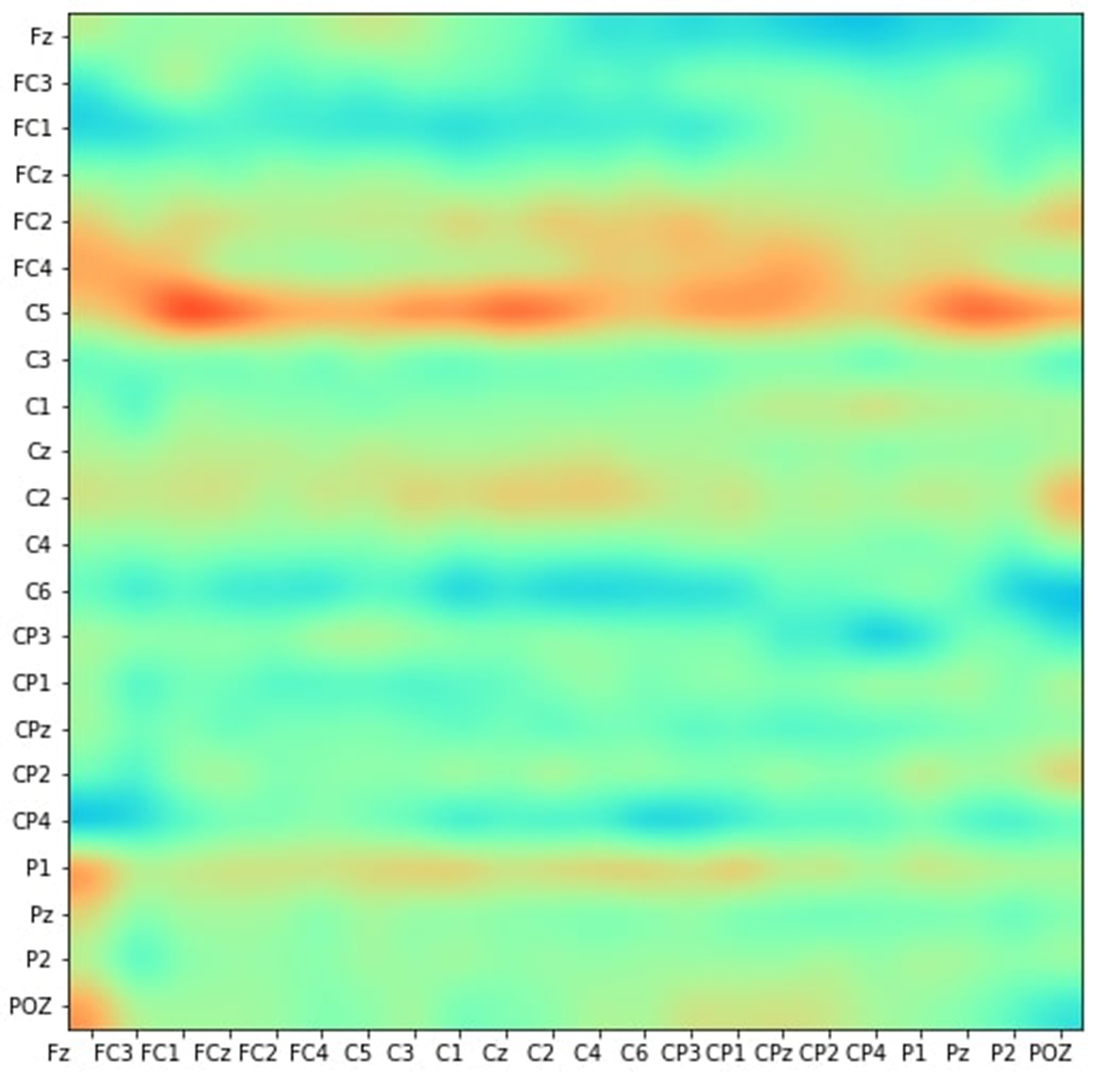

Supplement: Supplementary file 5 [file Image_5.JPEG]

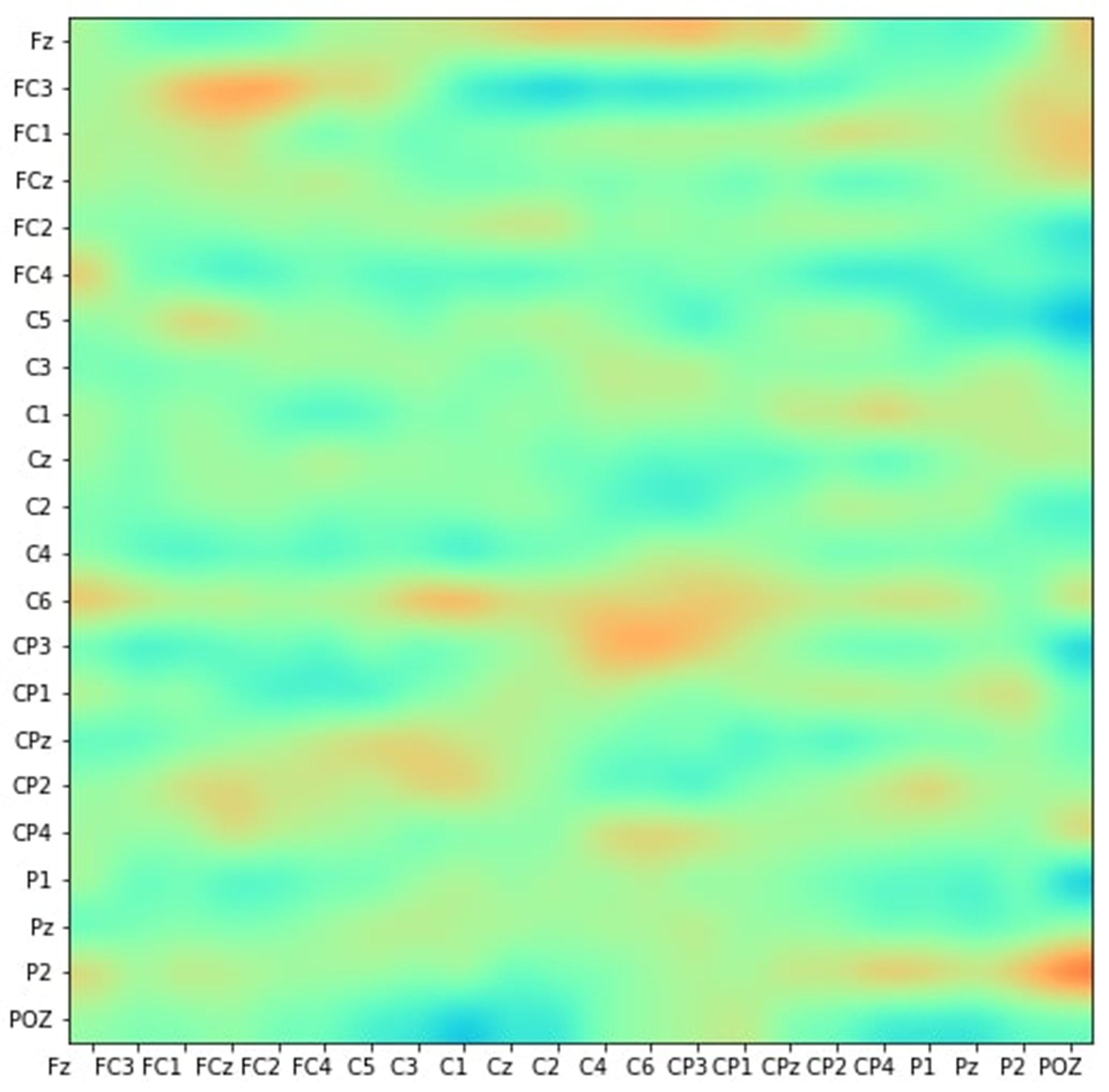

Supplement: Supplementary file 6 [file Image_6.JPEG]

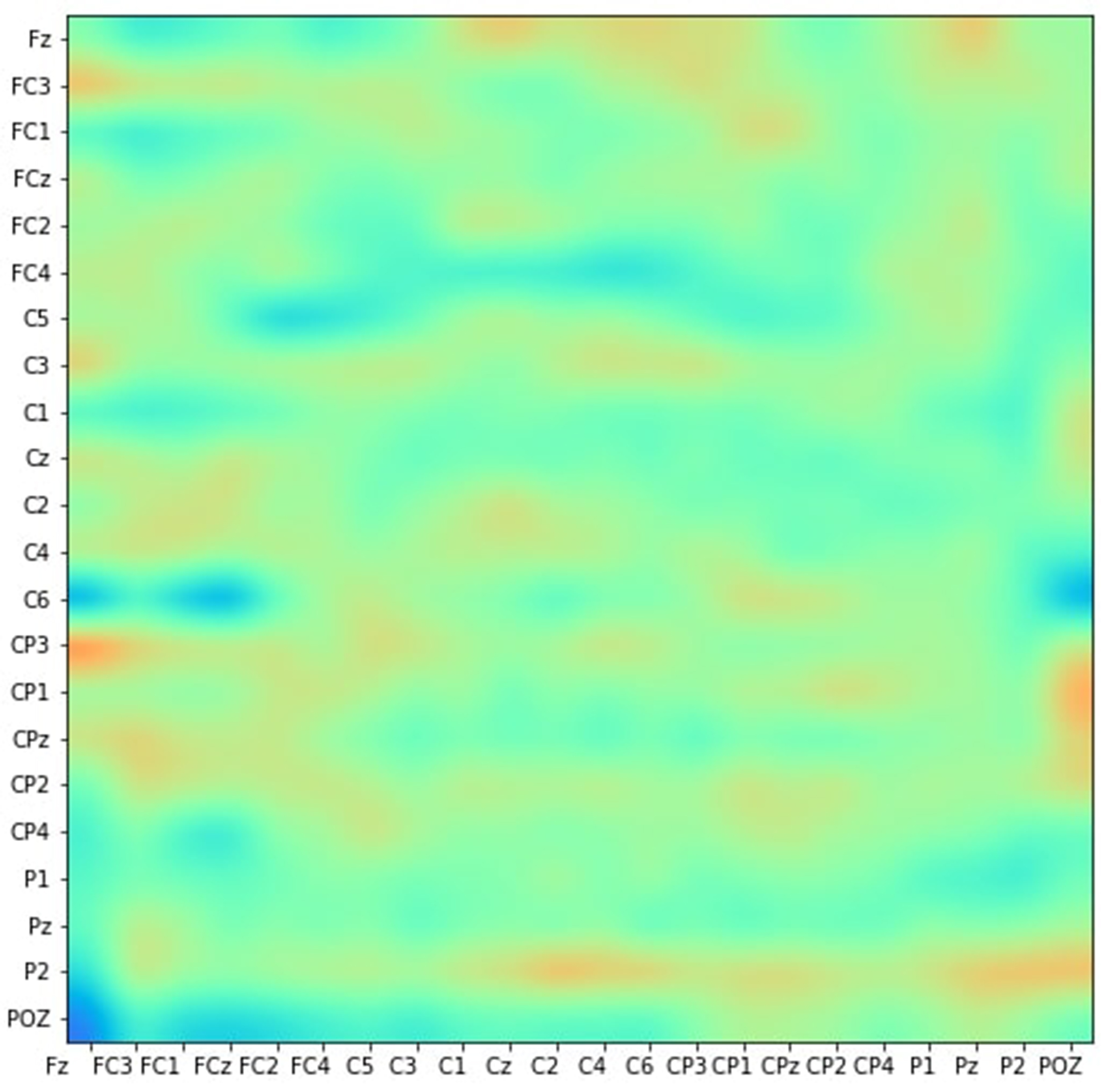

Supplement: Supplementary file 7 [file Image_7.JPEG]

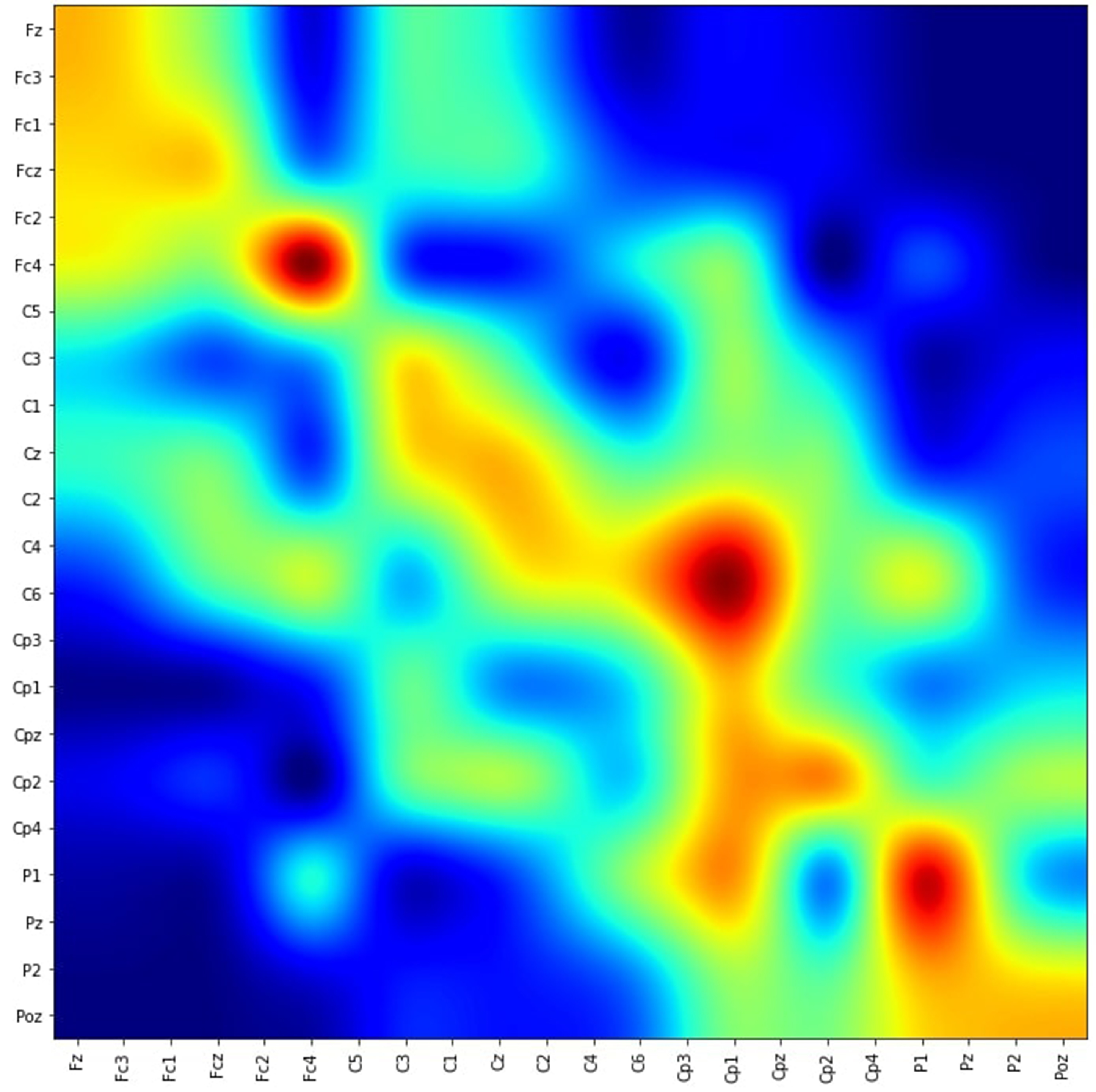

Supplement: Supplementary file 8 [file Image_8.JPEG]

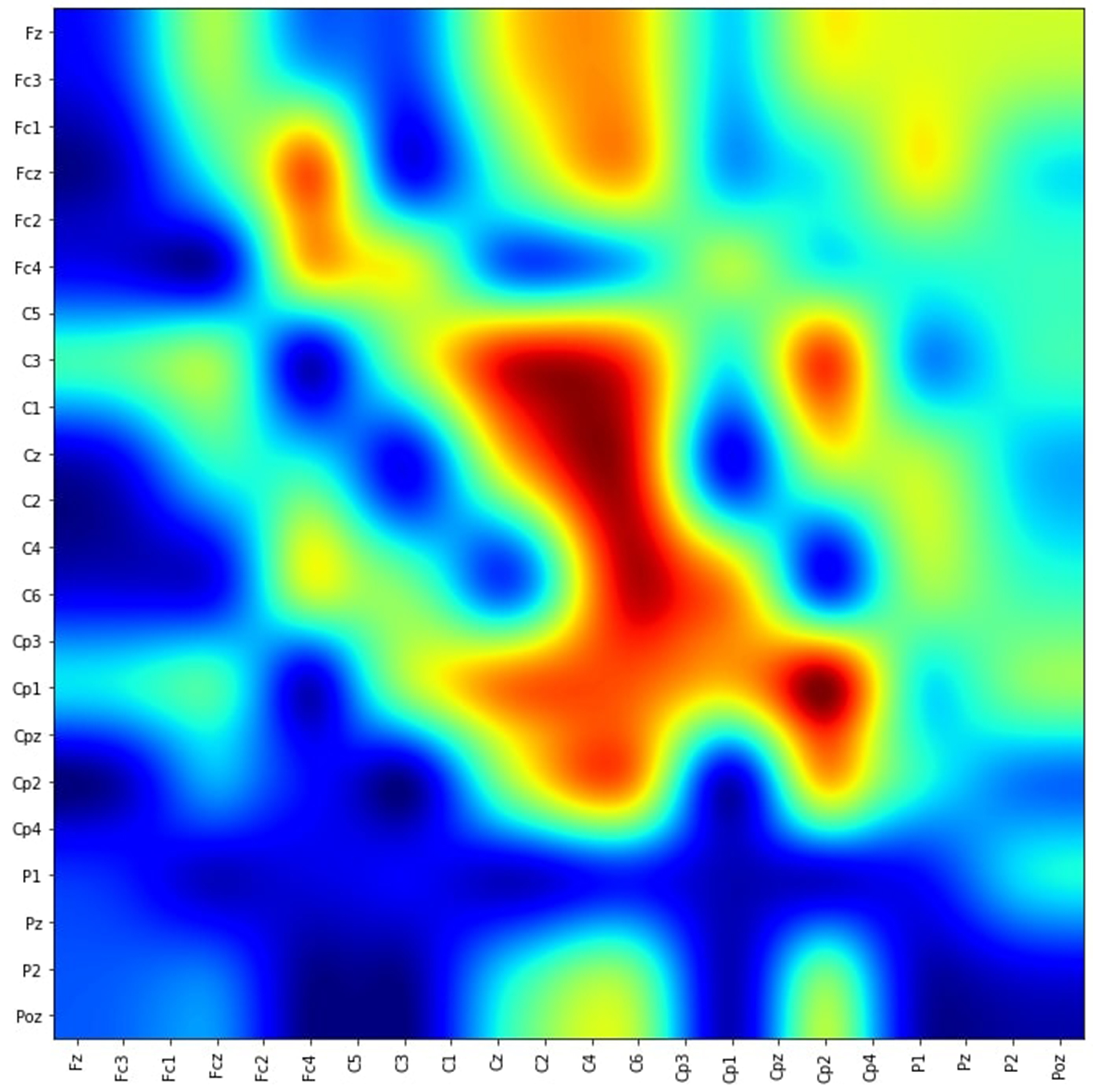

Supplement: Supplementary file 9 [file Image_9.JPEG]
